# Anti-Her2 affibody-decorated arsenene nanosheets induce ferroptosis through depleting intracellular GSH to overcome cisplatin resistance

**DOI:** 10.1186/s12951-023-01963-7

**Published:** 2023-06-27

**Authors:** Peng He, Shenglin Xu, Zhaohua Miao, Yukang Que, Yu Chen, Sheng Li, Qiming Ma, Rui Yang, Wei Wei, Zhengbao Zha, Yong Hu

**Affiliations:** 1 Department of Orthopedics, The First Affiliated Hospital of Anhui Medical University, Hefei, 230022 Anhui China; 2 School of Food and Biological Engineering, Hefei University of Technology, Hefei, 230009 Anhui China; 3 Department of Pharmacy, Anqing Medical College, Anqing, 246052 Anhui China; 4 Institute of Clinical Pharmacology, Anhui Medical University, Hefei, 230032 Anhui China

**Keywords:** Ferroptosis, Arsenene nanosheets, Chemotherapy, Cisplatin resistance, Osteosarcoma

## Abstract

Ferroptosis, a form of regulated cell death induced by excessive accumulation of reactive oxygen species and lipid peroxidation, has recently attracted extensive attention due to its ability to effectively suppress tumors and overcome drug resistance. Unlike previously reported metal nanomaterials that induce ferroptosis via the Fenton reaction, arsenene nanosheets can effectively deplete intracellular glutathione and then induce ferroptosis by inhibiting glutathione peroxidase 4. In this study, we designed target-modified arsenene nanosheets loaded with cisplatin (Her2-ANs@CDDP), which are capable of selective uptake by tumor cells. Her2-ANs@CDDP promotes both apoptosis and ferroptosis through a reciprocal cascade reaction between cisplatin and the carrier, respectively, and we demonstrate that it can significantly inhibit the activity of drug-resistant cells. Arsenene nanosheets kill drug-resistant tumor cells by inducing ferroptosis and restoring the sensitivity of drug-resistant cells to cisplatin. Cisplatin-loaded arsenene nanosheets can be prepared simply, and exert synergistic effects that overcome drug resistance. They show great potential for applications in the clinical treatment of chemotherapy-insensitive osteosarcoma, expanding the uses of arsenic in the treatment of solid tumors.

**Graphical abstract:**

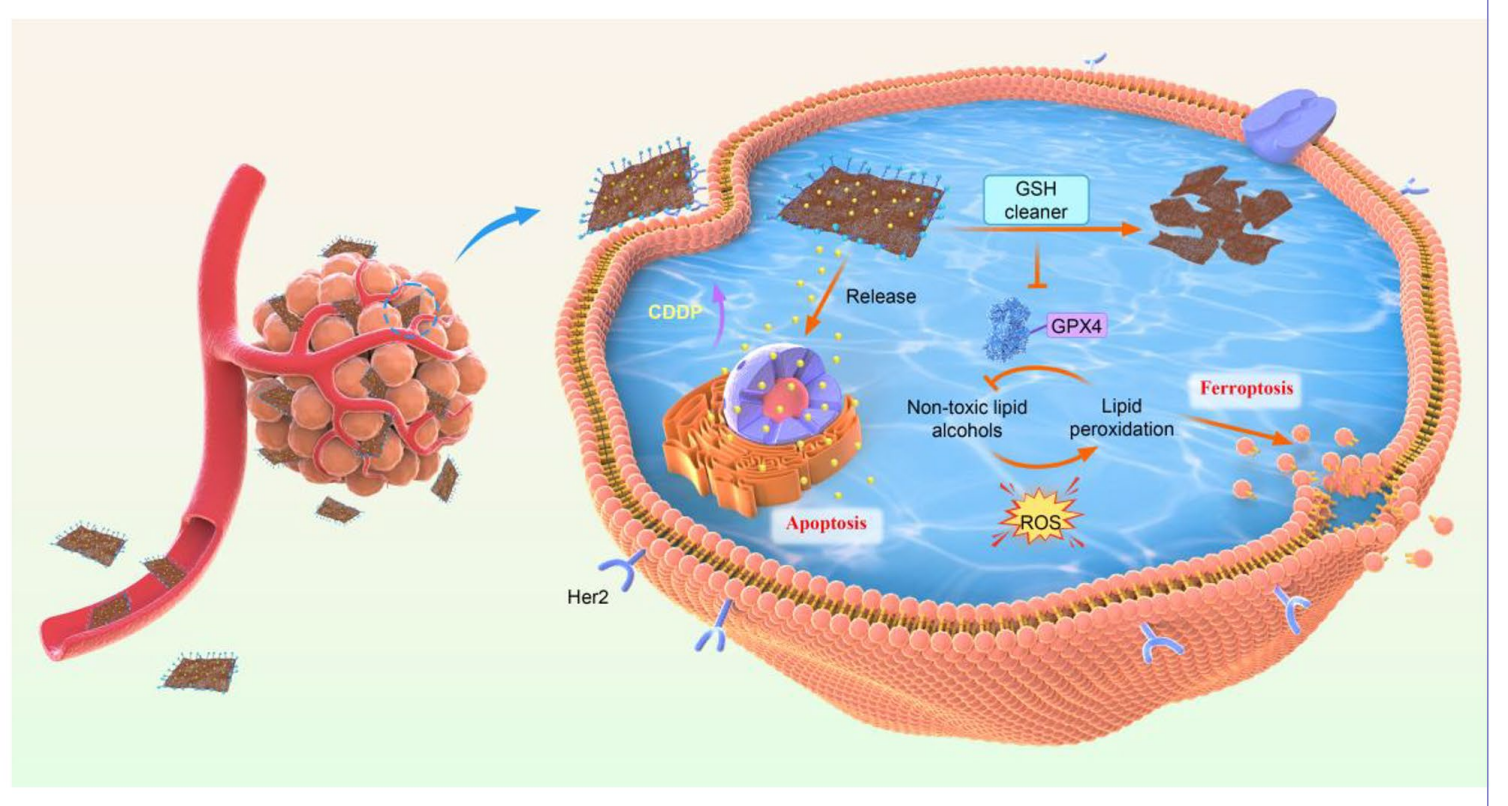

**Supplementary Information:**

The online version contains supplementary material available at 10.1186/s12951-023-01963-7.

## Introduction

Osteosarcoma (OS) is the most common primary malignant bone tumor, with approximately 70–80% of patients being children and adolescents [1]. The standard treatment protocol for OS is surgery combined with multi-course, multi-drug sequential neoadjuvant chemotherapy [2]. Although surgery combined with neoadjuvant chemotherapy can improve the limb salvage rate and survival rate of patients, patients are often forced to undergo amputation due to the emergence of chemotherapy resistance. OS can acquire chemoresistance through the expansion of chemotherapy-resistant cell populations, which are also responsible for tumor recurrences and metastases [3]. Therefore, it is essential to develop new therapies that can overcome chemotherapy resistance.

The first-line chemotherapy drugs for OS mainly induce caspase-dependent apoptosis [4]. However, some tumors do not respond to initial chemotherapy owing to intrinsic drug resistance, while other tumors that show an initial response often progress to drug resistance, ultimately leading to treatment failure. The mechanism of chemotherapy resistance is closely related to the self-protection of tumor cells, including reduced intracellular drug accumulation, increased drug detoxification, and increased DNA damage repair to combat chemotherapy-induced apoptosis [4, 5]. Therefore, other non-apoptotic regulatory cell death modalities have attracted increasing attention.

Ferroptosis is defined as a regulated cell death caused by rupture of the cell plasma membrane due to excessive accumulation of reactive oxygen species (ROS) and lipid peroxidation [6]. Because it is morphologically, biochemically, and genetically regulated differently from apoptosis, it offers a new therapeutic option for overcoming resistance to apoptosis [7]. The use of nanomaterials to induce ferroptosis is an emerging anti-tumor strategy [8]. Various iron-based nanomaterials have been shown to induce ferroptosis, potentially acting through elevating intracellular iron ions [9–11]. However, current iron-based nanomaterials usually require very high iron doses to induce ferroptosis, and the long-term safety of metal accumulation in vivo cannot be guaranteed [10]. Inactivation of the glutathione peroxidase 4 (GPX4)-related signaling pathway can also induce ferroptosis [12]. Glutathione peroxidase 4, which requires glutathione (GSH) as a cofactor, can reduce toxic phospholipid hydroperoxides (PLOOH) to non-toxic phosphatidyl alcohols, thus protecting cell membranes from rupture due to the accumulation of PLOOH [13]. Therefore, depletion of intracellular GSH can induce ferroptosis by inhibiting the activity of GPX4 [14]. Notably, there are no relevant reports on nonmetallic single-element nanomaterials that induce ferroptosis by depleting intracellular GSH.

Arsenic trioxide has demonstrated excellent efficacy in the treatment of newly diagnosed and relapsed acute promyelocytic leukemia [15]. Arsenic has a strong affinity for the sulfhydryl group of cysteine and much of its biological activity is mediated by inactivation of various sulfhydryl-containing enzymes and proteins [16–18]. The tripeptide glutathione, which contains sulfhydryl and cysteine and is widely distributed in cells, is a natural candidate to react with arsenic [13]. Moreover, the reducing and detoxifying effects of GSH are mainly dependent on sulfhydryl groups [19]. We hypothesize that arsenic has the potential to induce ferroptosis through the depletion of intracellular GSH.

The lack of tumor selectivity in traditional chemotherapy often results in inadequate accumulation of drugs at the tumor site and systemic side effects, which seriously affects the efficacy and safety of chemotherapy [1, 20]. Two-dimensional nanomaterials have a large surface area and unique surface chemistry, which allows them to be loaded with peptides and chemotherapeutic agents in a covalent or non-covalent manner [15, 21, 22]. The targeted modification of arsenene nanosheets (ANs) can achieve specific release and accumulation of chemotherapy drugs in tumor cells through active targeting and passive accumulation (enhanced permeability and retention effect, EPR) [23].

Many studies have identified proteins that are abundantly expressed on the surface of osteosarcoma cells, sparking ongoing targeting studies for these proteins [24]. Her2 is expressed in approximately 40% of osteosarcomas, making it a potential target for trastuzumab in the treatment of osteosarcoma [2, 25]. However, in a phase II clinical trial, it was demonstrated that combining trastuzumab with chemotherapy in Her2-positive osteosarcoma did not improve patient survival [26]. The development of targeted drug delivery based on the Her2 receptor on the surface of osteosarcoma cells might be expected to achieve better therapeutic results. Thus, appropriately designed arsenic-based nanomaterials might be capable of both targeting drug delivery to Her2-expressing cells and exerting intrinsic anti-tumor activity while enhancing chemotherapy and ferroptosis-mediated therapeutic effects.

In this study, ANs capable of inducing ferroptosis by inactivating GPX4 were successfully prepared using a liquid-phase exfoliation technique. We test the ability of Her2-target-modified cisplatin-loaded ANs to suppress the activity of cisplatin-resistant osteosarcoma cells and their effects on osteosarcoma in vivo using a mouse model.

## Result and discussion

### Validating the expression of Her2 in OS

**Fig. 1:**
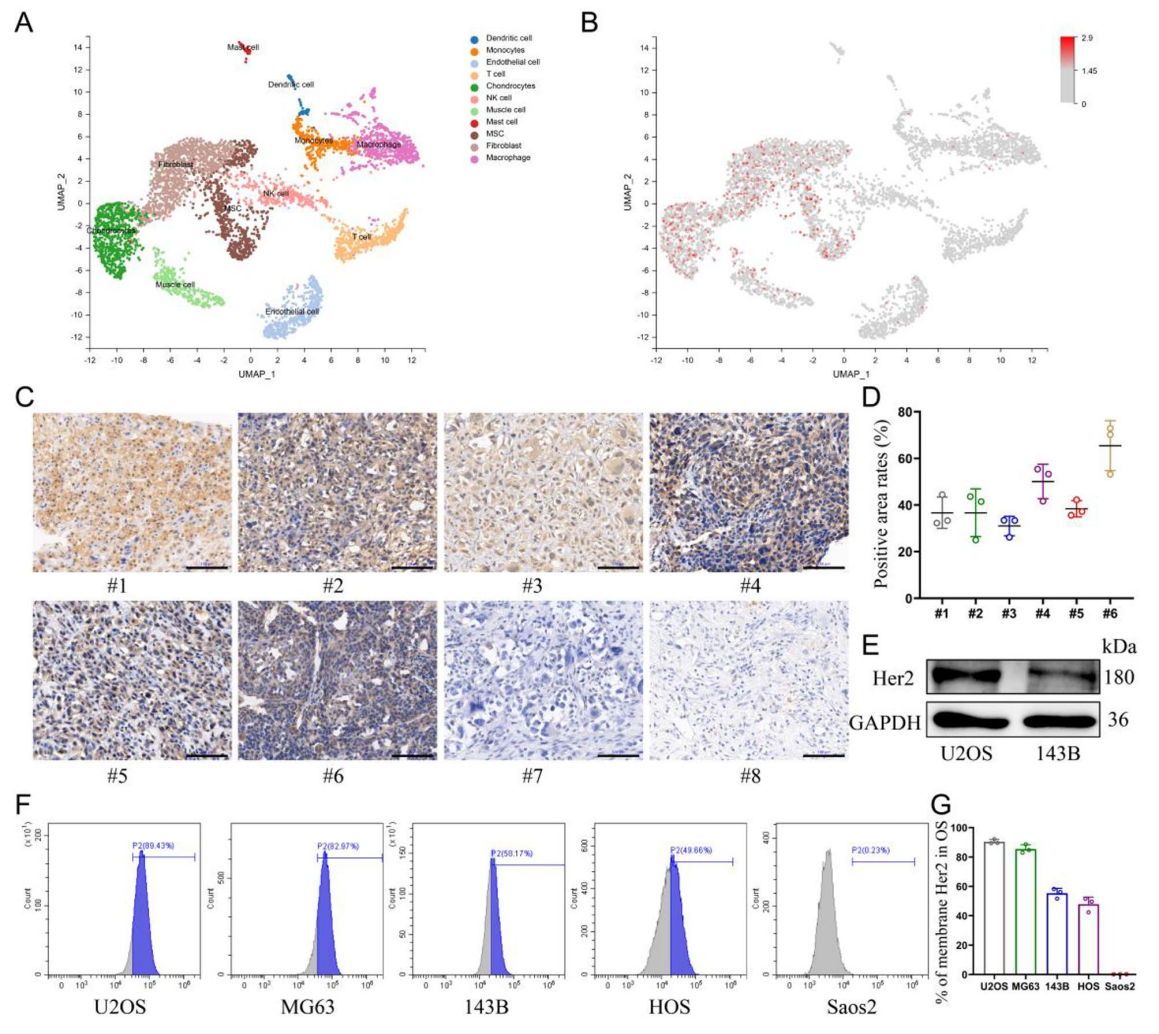
Validating the expression of Her2 in OS. **(A)** UMAP visualization of cells analyzed by scRNA-seq and integrated across 5 primary osteosarcomas. Clusters were annotated for their cell types as predicted using canonical markers. **(B)** Log-normalized expression of ERBB2(Her2). **(C)** The expression of Her2 in osteosarcoma derived from different patients assessed by immunohistochemistry assay. Scale bar: 100 μm. **(D)** Percentage of Her2-positive areas per osteosarcoma tissue. **(E)** Expression of Her2 in U2OS and 143B cells determined by Western blot. **(F, G)** The expression of Her2 in various osteosarcoma cell lines was detected by flow cytometry

The distribution of Her2 expression in osteosarcoma was explored by single-cell sequencing, which showed that Her2 was only aberrantly expressed in cell populations of malignant origin (Fig. 1A, B). Therefore, the selection of Her2 as a target would be expected to improve the tumor-homing capacity of nanomaterials and reduce the toxicity to normal tissues. Moreover, the expression levels of Her2 in osteosarcoma tissues from different patients were also distinct (Additional file 1: Fig. S1). Immunohistochemical analysis of samples collected from eight patients with OS revealed that six of them displayed Her2 expression in tumor tissues (Fig. 1C, D). Five human-derived OS cell lines were analyzed for Her2 expression by flow cytometry, and the results showed that four of them were positive (Fig. 1F, G). The results of western blotting showed a higher abundance of Her2 expression in U2OS cells than in 143B cells, which was also consistent with the results of flow cytometry (Fig. 1E).

### Synthesis and characterization of Her2-ANs

**Scheme 1:**
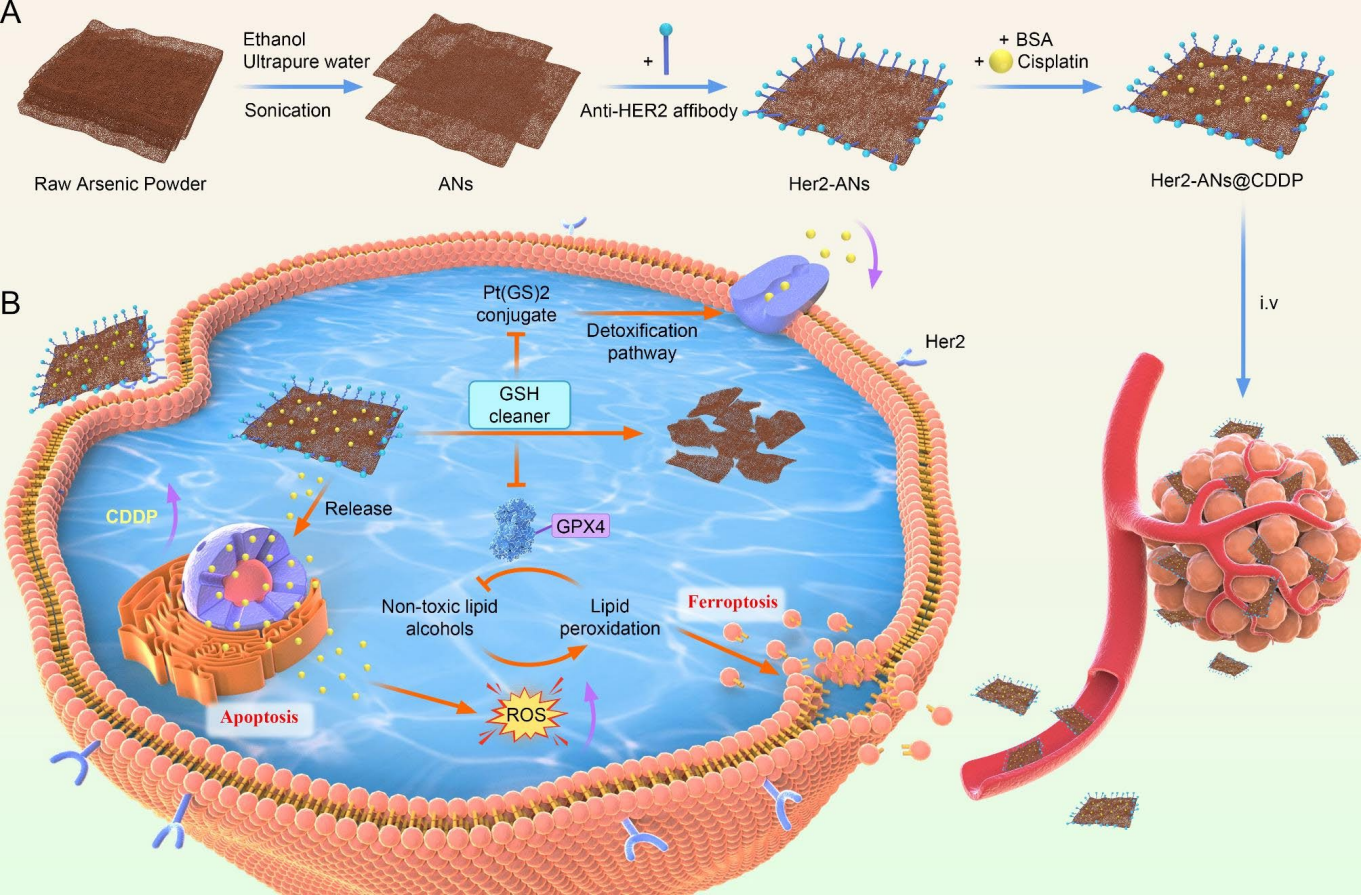
Schematic illustration of Her2-ANs@CDDP synthesis and arsenene nanosheets-induced ferroptosis

With the emergence of nanomedicine, there has been rapid development in the application of nanomaterials to the diagnosis and treatment of tumors [20]. However, the result of clinical translation is that only a few nanomaterials that can be applied to clinical oncology treatments. The main concerns are about the intrinsic biosafety of materials and the side effects caused by the abnormal accumulation of materials in vivo [10]. In 1996, it was demonstrated that arsenic trioxide as a single agent could induce complete remission of acute promyelocytic leukemia with only mild myelosuppression [16, 27]. However, the therapeutic effect of arsenic trioxide in solid tumors is far from satisfactory due to the lack of targeting [18]. In this study, we have successfully prepared ANs by a liquid phase exfoliation technique. The complete synthetic route of ANs is shown in Scheme 1 A.

**Fig. 2:**
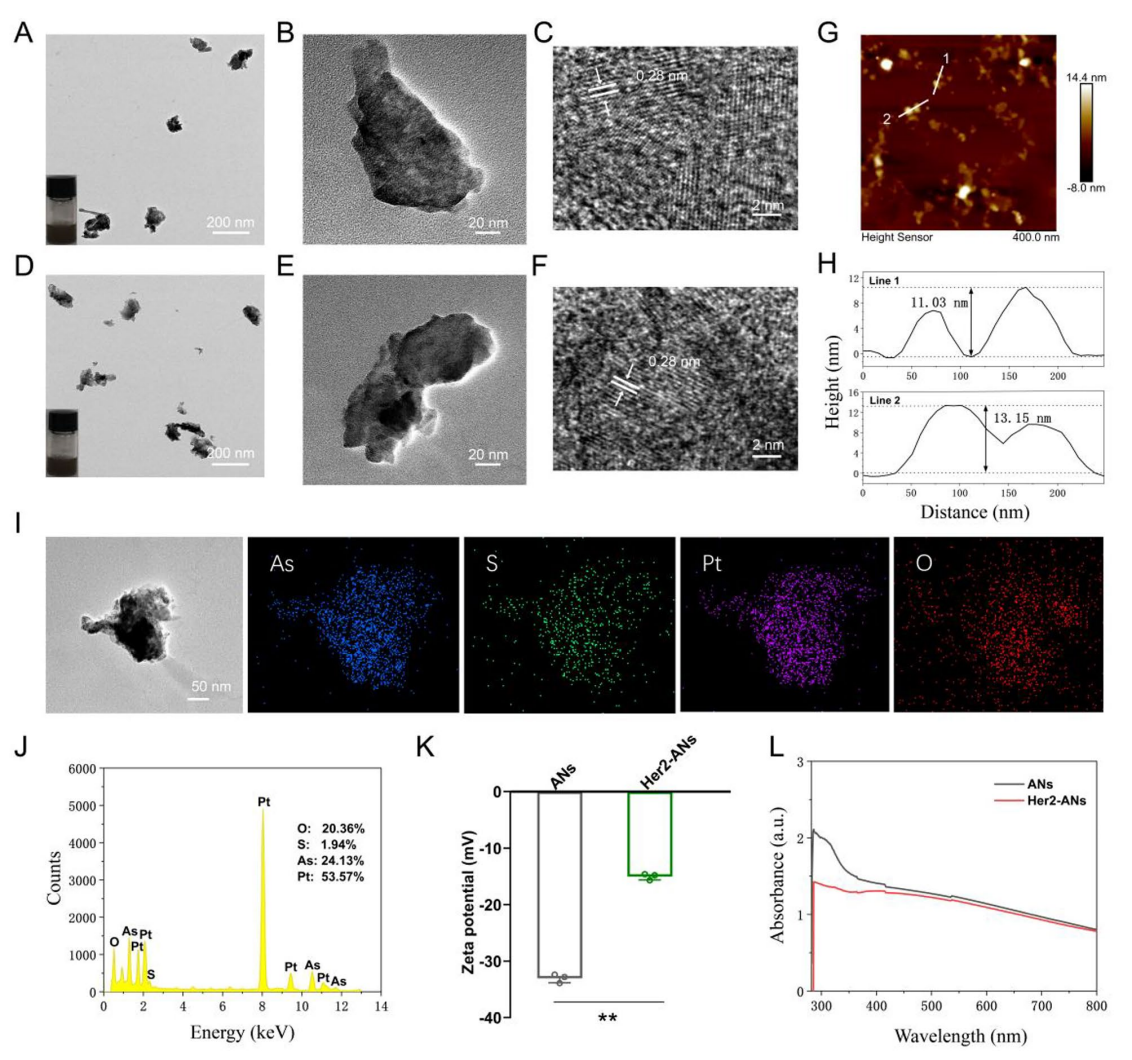
Characterization of the ANs. **(A)** TEM image of ANs, and photograph of ANs in solution (inset). **(B, C)** HRTEM images of ANs. **(D)** TEM image of Her2-ANs, and photograph of Her2-ANs in solution (inset). **(E, F)** HRTEM images of Her2-ANs. **(G)** AFM image of Her2-ANs. **(H)** Corresponding height profiles along the white lines. **(I, J)** The corresponding elemental mapping **(I)** and EDS **(J)** of Her2-ANs@CDDP. **(K)** Zeta potentials of ANs and Her2-ANs. **(L)** UV − vis spectra of ANs and Her2-ANs. (Values are presented as means ± sd, n = 3, *P < 0.05, **P < 0.01.)

Transmission electron microscope (TEM) images show that the ANs exhibit ultrathin nanosheet morphology (Fig. 2B). The TEM images reveal that the size of ANs is about 131 nm (Fig. 2B), which is significantly different from the raw arsenic powder with a size larger than 2 μm (Additional file 1: Fig. S3A). The morphology and height of the raw arsenic powder and Her2-ANs were analyzed by AFM, and the results showed that the height of the nanosheets was about a dozen nanometers (Fig. 2H), which was significantly different from the raw arsenic powder with a height greater than 2 μm (Additional file 1: Fig. S3C). In addition, the difference in absorbance between raw arsenic powder, ANs and Her2-ANs was analyzed by UV-Vis absorption spectroscopy. The absorbance of the raw arsenic powder increased gradually between 300 and 800 nm (Additional file 1: Fig. S3D), whereas the absorbance of ANs and Her2-ANs decreased significantly in this range (Fig. 2L). The high-resolution transmission electron microscopy (HRTEM) images show the presence of 0.28 nm lattice fringes (Fig. 2C, F), which are characteristic of crystalline material. The crystal structures of the raw arsenic powder and ANs were further analyzed by Raman spectroscopy (Additional file 1: Fig. S4B, C). The Raman spectra showed two characteristic peaks at 209.6 and 242.2 cm^-1^ for the raw arsenic powder, which was different from the characteristic peaks for the ANs (211.4 and 244 cm^-1^). As the density of anhydrous ethanol is lower than that of ultrapure water, anhydrous ethanol can isolate oxygen during the process of exfoliation. There were also no characteristic peaks for arsenic oxide in the Raman spectrum. Moreover, ethanol can lower the temperature of the synthesis system through volatilization, which can increase the yield of nanosheets [15]. All the above results confirm the successful exfoliation of ultrathin ANs from the raw arsenic powder. Digital photographs of arsenic in ultrapure water (Fig. 2A, D; insets) show that the ANs and Her2-ANs are homogeneously dispersed in solution.

To achieve active targeting of the nanosheets, an Anti-Her2 Affibody was linked to the surface of ANs. Anti-Her2 Affibody is a peptide with a C-terminal cysteine residue, which can be covalently bound to arsenic [28]. The mode of action of arsenic trioxide in the treatment of promyelocytic leukemia is based on the direct binding of arsenic to the cysteine residues of the zinc finger located in the promyelocytic leukemia protein [29]. When Anti-Her2 Affibody was linked to ANs, the zeta potential changed from − 33mV to -15mV, which is consistent with the results of two previous affibody modification studies (Fig. 2K) [30, 31]. Since the molecular size of the affibody was only 14 kDa, the size of the nanosheets increased slightly after the affibody modification (Additional file 1: Fig. S4F). FTIR spectra show characteristic absorption bands of ANs and Her2-ANs at different frequencies. After affibody modification, the absorption bands were changed at 1650 cm^-1^ and 1366 cm^-1^ (Additional file 1: Fig. S4A) [30]. The elemental composition of Her2-ANs was analyzed using energy dispersive spectroscopy (EDS) and the results show that elemental sulfur was uniformly distributed in Her2-ANs after connection of the affibody (Additional file 1: Fig. S4D). In addition, a slight shift in the characteristic peaks of Her2-ANs after affibody attachment was found by comparing the Raman spectra of ANs and Her2-ANs (Additional file 1: Fig. S4B). These results confirmed that the Anti-Her2 Affibody was successfully connected to the surface of ANs.

### Drug loading and release

**Fig. 3:**
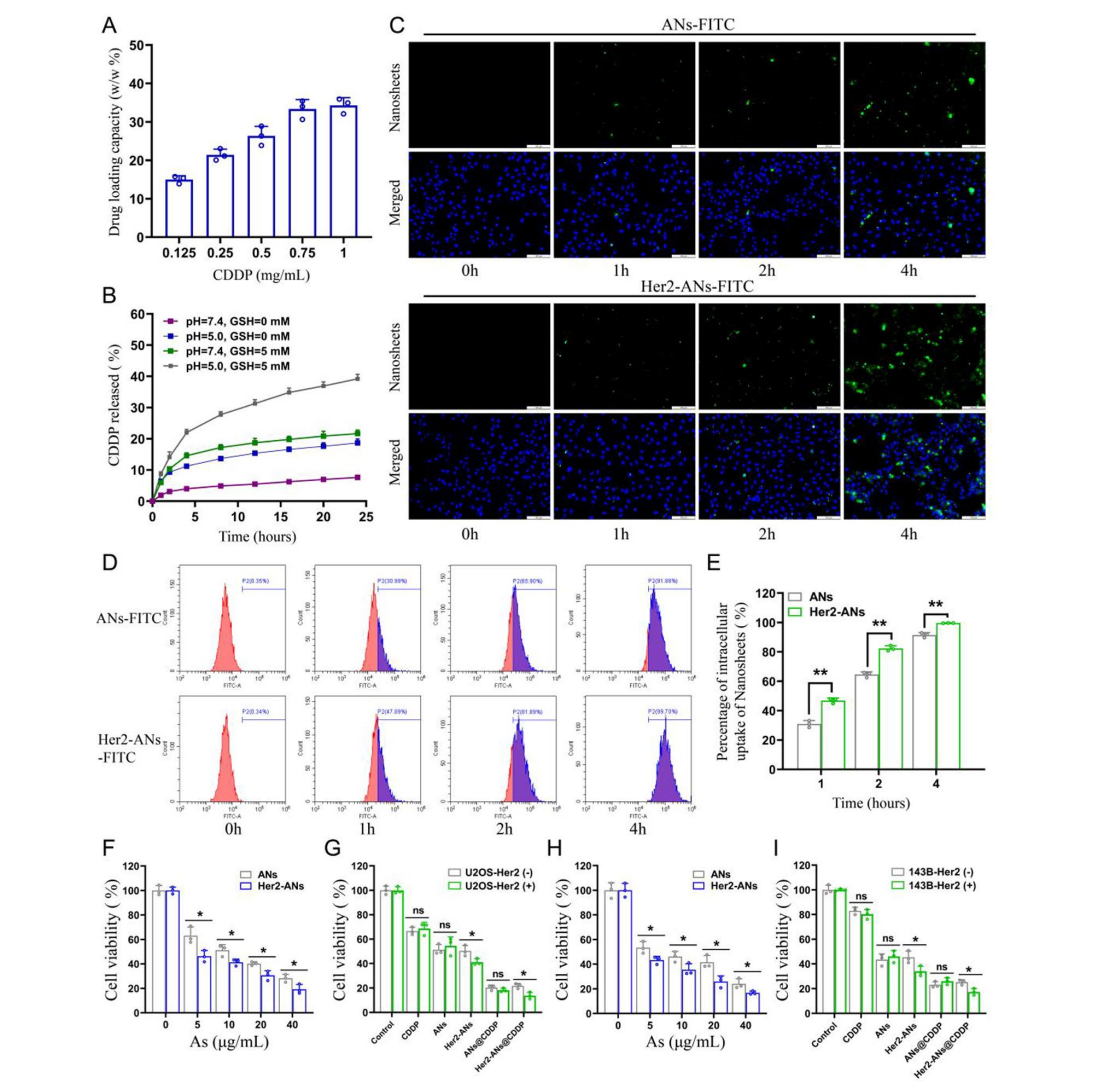
In vitro tumor cell uptake and cytotoxicity. **(A)** CDDP loading capacities of Her2-ANs. **(B)** Release profiles of Her2-ANs@CDDP at different pH values and different GSH concentrations. **(C)** Intracellular uptake of ANs and Her2-ANs (labeled with FITC) in the U2OS-Her2(+) cells was measured by confocal laser scanning microscopy. Scale bar: 100 μm. **(D)** Intracellular uptake of ANs and Her2-ANs (labeled with FITC) in the U2OS-Her2(+) cells was measured by flow cytometry. **(E)** Statistical analyses of the intracellular uptake rate of ANs and Her2-ANs. **(F, G)** Cytotoxicity of ANs and Her2-ANs against U2OS-Her2(+) cells **(F)**. Relative viabilities of U2OS-Her2(+) and U2OS-Her2(-) cells after incubation with various treatments **(G)** (CDDP: 2 µg/mL; As ions: 10 µg/mL). **(H, I)** Cytotoxicity of ANs and Her2-ANs against 143B-Her2(+) cells **(H)**. Relative viabilities of 143B-Her2(+) and 143B-Her2(-) cells after incubation with various treatments **(I)** (CDDP: 1 µg/mL; As ions: 10 µg/mL). (Values are presented as means ± sd, n = 3, *P < 0.05, **P < 0.01.)

Single-element ANs as a two-dimensional nanomaterial may be a promising carrier for chemotherapy drugs [15]. Results from inductively coupled plasma (ICP) showed that the maximum cisplatin loading rate of Her2-ANs was 34.3 w/w%, which is significantly higher than the approximately 5–15% cisplatin loading rate reported in previous nanocarrier-related studies (Fig. 3A) [32, 33]. Based on the results of preliminary experiments, the ANs could not be loaded with cisplatin without BSA modification, which indicates that BSA assists in cisplatin loading. After 24 h of cisplatin intravenous infusion, approximately 80% of cisplatin is bound to plasma proteins, in particular to serum albumin [34]. It was demonstrated in vitro that cisplatin binding to bovine serum albumin (BSA) occurs mainly through coordination with histidine or methionine side chains [35, 36]. In addition, the EDS mapping showed that S and Pt were uniformly distributed in Her2-ANs@CDDP and the contents of As, S, O and Pt were 24.13%, 1.94%, 20.36% and 53.57%, respectively (Fig. 2I, J).

Many of the biological effects of arsenic are mediated by the inactivation of various sulfhydryl-containing enzymes or proteins in cells, and sulfhydryl-containing GSH is known to be widely distributed in cells [13, 18]. Therefore, we hypothesized that arsenic would effectively deplete intracellular GSH. Firstly, we evaluated the GSH scavenging capacity of Her2-ANs in vitro. After incubating of Her2-ANs with GSH for 12 h, the concentration of GSH was lowered by 67% (Additional file 1: Fig. S6). Based on these results, we further observed the degradation of Her2-ANs by TEM under various GSH and pH conditions. In the absence of GSH, the morphology of Her2-ANs remained almost unchanged (Additional file 1: Fig. S5). However, the edges of the nanosheets became blurred after 12 h of incubation in an environment with GSH (5 mM). After 24 h, the nanosheets were observed to degrade into smaller-sized nanofragments (Additional file 1: Fig. S5).

After determining the stability of ANs under different environments, we further explored the stimulus-responsive release ability of Her2-ANs@CDDP under different pH and GSH conditions. In the conditions without GSH (pH = 7.4), Her2-ANs@CDDP released only 7.59% of cisplatin for 24 h, indicating that the nanocarriers were relatively stable before reaching the tumor microenvironment (Fig. 3B). The release rate of cisplatin could be increased to 21.65% at 24 h by increasing the GSH concentration (pH = 7.4, GSH = 5 mM), which could be attributed to the nanosheets decomposing in the presence of GSH. The release rate of cisplatin could be increased to 18.67% at 24 h by lowering the pH (pH = 5.0, GSH = 0 mM) [32, 37]. In a simulated tumor microenvironment (pH = 5.0, GSH = 5 mM), the cumulative release of Her2-ANs@CDDP could reach 39.18% at 24 h (Fig. 3B). Considering that the tumor microenvironment is characterized by high GSH content and low pH, Her2-ANs@CDDP can selectively release drugs into the interior of the tumor, which can reduce the side effects of cisplatin [13, 30].

### In vitro tumor cell selective uptake and cytotoxicity

Since Her2 expressed on cell membranes is recognized specifically by Anti-Her2 Affibody, the affibody may contribute to the endocytosis of Her2-ANs [30]. We performed cellular uptake studies by laser confocal microscopy, flow cytometry and ICP-MS. For U2OS-Her2(+) cells, the uptake of Her2-ANs-FITC was significantly higher than that of ANs-FITC due to the binding of Her2 and Anti-Her2 Affibody, which was mainly manifested by the stronger green fluorescent signal exhibited in the Her2-ANs-FITC group (Fig. 3C). Analysis by flow cytometry showed that the uptake rates of Her2-ANs-FITC in U2OS-Her2(+) cells reached 81.89% and 99.7% at 2 and 4 h, respectively, whereas the uptake rates of ANs-FITC were only 65.9% and 91.88% (Fig. 3D, E). Quantitative assessment of cellular uptake of arsenic material by ICP-MS showed that Her2-positive cells took up significantly more Her2-ANs than ANs at the same dose, which is consistent with the results of laser confocal microscopy and flow cytometry (Additional file 1: Fig. S8A). In addition, ICP-MS results showed that raw arsenic powders with sizes exceeding 2 μm were barely taken up by the cells.

The cytotoxicity induced by ANs was assessed by CCK-8 and cell death assays to verify whether the nanosheets could be applied to tumor therapy. Compared with the low toxicity to AML-12 normal hepatocytes, the viability of U2OS-Her2(+) and 143B-Her2(+) cells was below 20% after 20 h incubation with Her2-ANs (40 µg/mL), suggesting that arsenene nanosheets were able to selectively kill tumor cells (Fig. 3H and Additional file 1: Fig. S7C). As shown in Fig. 3F, H, the killing effect of Her2-ANs on Her2-positive tumor cells was significantly higher than that of ANs, which was mainly attributed to the increased cellular uptake of nanosheets after coupling them with Her2. In addition, CCK-8 and cell death assays showed that the antitumor effect of Her2-ANs@CDDP was the best among all treatment groups, suggesting a synergistic antitumor effect of ANs and cisplatin (Fig. 3G, I and Additional file 1: Fig. S7B). Collectively, these results suggest that smaller sizes, sheet structures and targeted modifications are essential for the uptake and cytotoxic effects of arsenic materials.

### Mechanism underlying ANs-induced ferroptosis

**Fig. 4:**
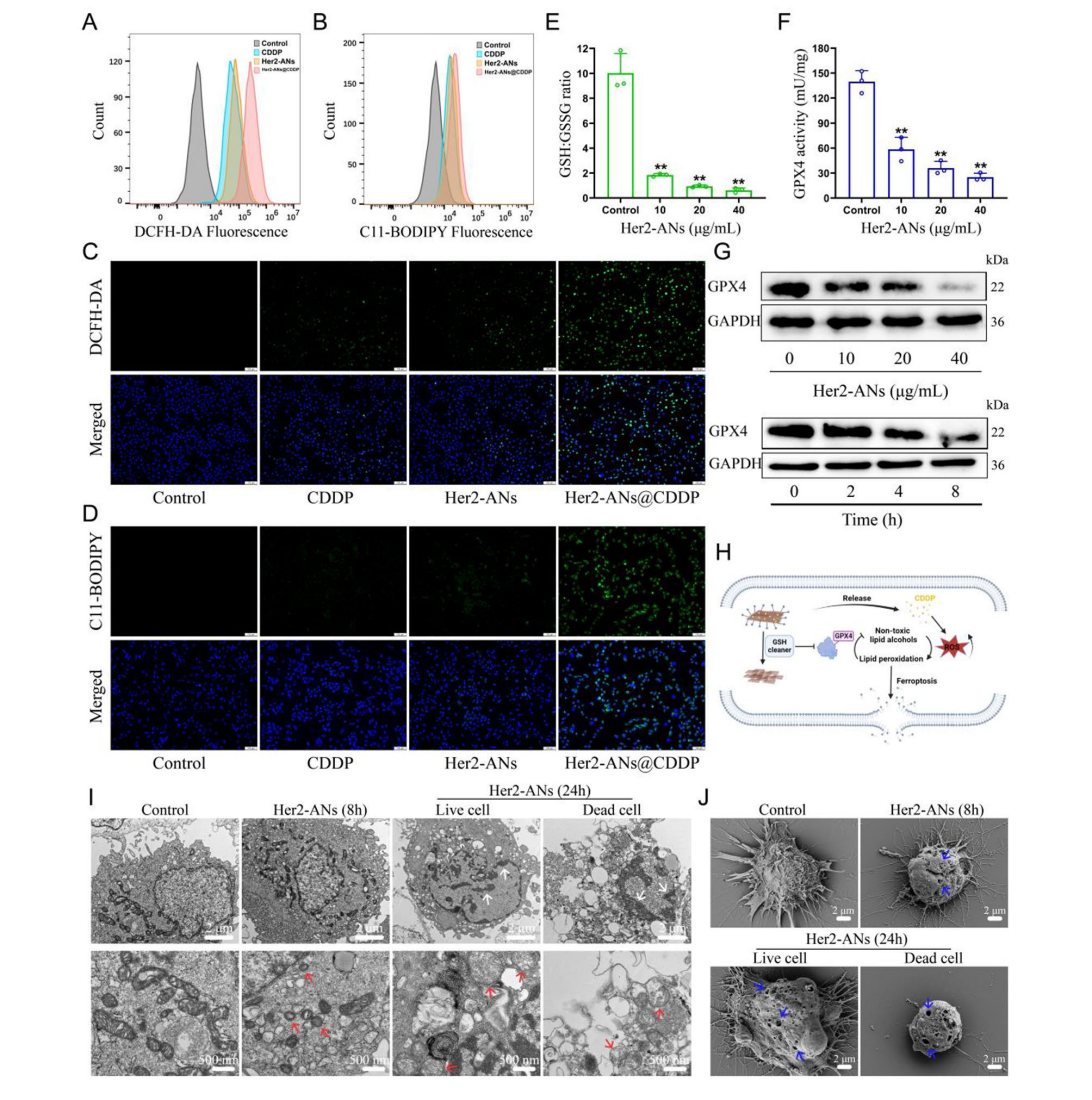
Mechanism evaluation of ANs induced cell death by ferroptosis. **(A, B)** Flow cytometry analysis of the production of ROS **(A)** and lipid peroxide **(B)** in U2OS-Her2(+) cells after different treatments. **(C, D)** Confocal laser scanning microscopy imaging of the production of ROS **(C)** and lipid peroxide **(D)** levels in U2OS-Her2(+) cells after different treatments. Scale bar: 100 μm. **(E, F)** GSH: GSSG ratio **(E)** and GPX4 activity **(F)** in U2OS-Her2(+) cells treated with Her2-ANs. **(G)** Western blot results for GPX4 expression in U2OS-Her2(+) cells after treatment with Her2-ANs. **(H)** Schematic illustration of Her2-ANs induced ferroptosis. Arsenene nanosheets could effectively deplete intracellular GSH and then induce ferroptosis by inhibiting GPX4. **(I, J)** TEM **(I)** and SEM **(J)** reveal nuclear, mitochondrial, and cell membrane alterations in U2OS-Her2(+) cells after treatment with Her2-ANs. (The white arrow represents DNA damage, the red arrow represents mitochondria, and the blue arrow represents cell membrane damage.)

Considering the outstanding antitumor activity and GSH scavenging ability of ANs, we hypothesized that arsenene nanosheets could induce ferroptosis by depleting intracellular GSH and inhibiting the activity of GPX4. Hence, we tested the ability of Her2-ANs to deplete intracellular GSH. The results showed that the levels of intracellular GSH were significantly decreased after Her2-ANs treatment compared to the control group (Fig. 4E). Since activation of GPX4 requires GSH as a cofactor, depletion of intracellular GSH will affect the activity of GPX4 [38]. The expression level and activity of intracellular GPX4 decreased substantially with increasing concentration and incubation time of Her2-ANs (Fig. 4F, G).

The GSH-centered redox system is the intracellular oxidative defense barrier that can most effectively scavenge intracellular ROS [39]. According to the results from staining using the ROS probe (DCFH-DA), the level of ROS was significantly increased in Her2-ANs-treated cells, which was attributed to the depletion of intracellular GSH by Her2-ANs (Additional file 1: Fig. S9A, C). By activating nicotinamide adenine dinucleotide phosphate oxidase (NOX), cisplatin can produce large amounts of intracellular O_2_^·-^, which can be converted into H_2_O_2_ under the action of superoxide dismutase. Subsequently, H_2_O_2_ catalyzes the production of ROS in the cell through the Fenton reaction, which is consistent with the results of flow cytometry and laser confocal microscopy images (Fig. 4A, C) [40, 41]. The stronger intracellular ROS fluorescence signal after Her2-ANs@CDDP treatment compared to cisplatin was attributed to the cisplatin-induced ROS production and reduced GSH with antioxidant activity. Excess intracellular ROS can lead to the accumulation of lipid peroxides, which is a key hallmark of ferroptosis [42]. In addition, intracellular lipid peroxides cannot be converted to non-toxic lipid alcohols due to the inhibition of GPX4 activity [38]. Similar to the results of ROS, the strongest intracellular lipid peroxide fluorescence signal was observed after Her2-ANs@CDDP treatment (Fig. 4B, D). These results suggest that the strategy of depleting GSH combined with ROS self-supplementation synergistically promote ferroptosis (Fig. 4H).

Morphologically, the accumulation of excess lipid peroxides increases membrane permeability and damages the cell membrane, eventually leading to the rupture of the cell membrane [7, 43]. The changes in cell membranes after treatment with Her2-ANs were observed by Scanning electron microscope (SEM). The results showed pore structures of varying sizes on the surface of the cell membrane, indicating a loss of cell membrane integrity (Fig. 4J). In addition, TEM imaging of Her2-ANs-treated cells revealed a marked atrophy of mitochondria accompanied by the disappearance of cristae, a typical morphological change of ferroptosis (Fig. 4I) [7]. After 24 h, chromatin condensation was also observed in cells treated with Her2-ANs, which may be attributed to DNA damage caused by persistent and extensive intracellular oxidative stress (Fig. 4I) [44]. Unlike previously reported metal nanomaterials that induce ferroptosis via the Fenton reaction, ANs, which are classified as inorganic non-metallic materials, can be used as a novel ferroptosis inducer by depleting intracellular GSH.

To further validate that ANs induce ferroptosis, regulated cell death was analyzed using several inhibitors associated with the ferroptosis pathway. As expected, ferroptosis inhibitor (Fer-1) and iron chelator (DFOM) prevented cell death induced by Her2-ANs, whereas apoptosis inhibitor (Z-VAD-FMK) did not have any effect (Additional file 1: Fig. S8B). In addition, the addition of antioxidants (GSH or NAC) significantly inhibited the cytotoxic effects of Her2-ANs. These results suggest that oxidative stress and ferroptosis play a key role in the cell death induced by Her2-ANs.

### ANs overcome cisplatin resistance by restoring intracellular drug accumulation

**Fig. 5:**
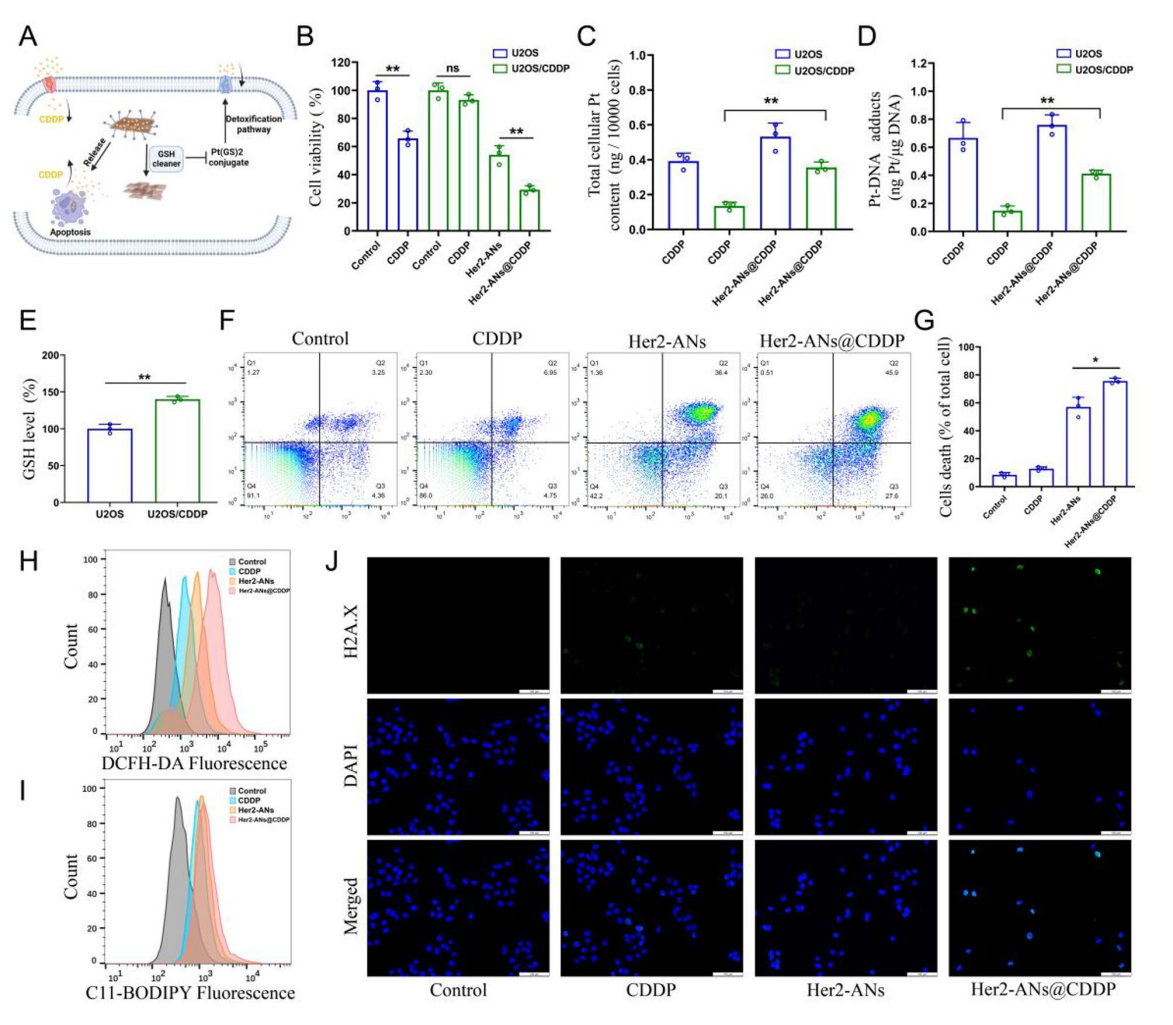
ANs overcome cisplatin resistance by restoring intracellular drug accumulation. **(A)** Schematic illustration of arsenene nanosheets restores the sensitivity of drug-resistant cells to cisplatin. **(B)** Relative viabilities of U2OS and U2OS/CDDP cells after various treatments (CDDP: 2 µg/mL; As ions: 10 µg/mL). **(C, D)** Total intracellular Pt content **(C)** and DNA-binding Pt content **(D)** in U2OS and U2OS/CDDP cells after incubation with CDDP or Her2-ANs@CDDP (CDDP: 2 µg/mL). **(E)** The level of GSH in U2OS and U2OS/CDDP cells. **(F, G)** Flow cytometry analysis of cell death in U2OS/CDDP cells treated with various treatments (CDDP: 2 µg/mL; As ions: 10 µg/mL). **(H, I)** Flow cytometry analysis of the production of ROS **(H)** and lipid peroxide **(I)** in U2OS/CDDP cells after different treatments. **(J)** Immunofluorescence staining of γH2A.X formation (green fluorescence) in U2OS/CDDP cells after various treatments (CDDP: 2 µg/mL; As ions: 10 µg/mL). Scale bar: 100 μm. (Values are presented as means ± sd, n = 3, *P < 0.05, **P < 0.01.)

Cisplatin is the first-line chemotherapeutic agent for OS, and the development of cisplatin resistance is a challenge for tumor treatment [45]. Ferroptosis is characterized by an imbalance in the intracellular redox state accompanied by elevated levels of ROS and lipid peroxides, which is a completely different mechanism to apoptosis [7, 46]. Inducing ferroptosis is one of the best ways to overcome apoptosis resistance [38]. To test whether Her2-ANs@CDDP can effectively overcome and reverse cisplatin resistance, we constructed cisplatin-resistant OS cells by stepwise drug screening. The IC50 values of cisplatin against U2OS and U2OS/CDDP cells were 2.437 µg/mL and 12.98 µg/mL, respectively, indicating the successful construction of drug-resistant cells (Additional file 1: Fig. S11). The results showed that Her2-ANs significantly inhibited the activity of U2OS/CDDP cells, whereas cisplatin did not exhibit any cytotoxic effect (Fig. 5B). Resembling the previous results, ROS and lipid peroxidation levels were significantly increased in Her2-ANs treated U2OS/CDDP cells (Fig. 5H, I). These results suggest that Her2-ANs can overcome the drug resistance of U2OS/CDDP cells by inducing ferroptosis.

Cisplatin resistance is closely associated with increased levels of intracellular GSH, and multidrug resistance-associated proteins (MRPs) are also involved in the development of cisplatin resistance [47, 48]. Cisplatin can bind GSH to form Pt(GS)2 conjugates before binding to DNA, and then Pt(GS)2 is excreted from the cell mediated by MRPs, leading to a decrease in intracellular content of the drug [5, 32]. Therefore, we measured GSH in U2OS/CDDP cells, and the results showed significantly higher levels of GSH in resistant cells compared to their parental cells (Fig. 5E). Based on the role of GSH in cisplatin resistance and the ability of ANs to deplete intracellular GSH, we hypothesized that ANs may reduce Pt(GS)2 formation by decreasing intracellular GSH, thereby increasing intracellular accumulation of cisplatin and restoring the sensitivity of U2OS/CDDP cells to cisplatin (Fig. 5A). As shown in Fig. 5F, the highest cell mortality was detected after Her2-ANs@CDDP treatment, indicating that U2OS/CDDP cells regained drug sensitivity.

To verify whether the elevated cytotoxicity was due to increased intracellular drug accumulation, we examined the levels of Pt in the cells and the DNA. Both total Pt and DNA-bound Pt were significantly increased in Her2-ANs@CDDP-treated cells compared to the cisplatin-treated group (Fig. 5C, D). Cisplatin-induced DNA damage results in the formation of γH2A.X at the site of damage, which is a sign of a DNA double-strand break [5]. Consistent with the analyses of Pt-DNA, immunofluorescence showed the strongest γH2A.X fluorescence signal in the Her2-ANs@CDDP treated group (Fig. 5J). These results indicate that ANs can not only kill drug-resistant tumor cells by inducing ferroptosis, but can also improve the sensitivity of drug-resistant cells to cisplatin.

### Assessment of in vivo antitumor efficacy

**Fig. 6:**
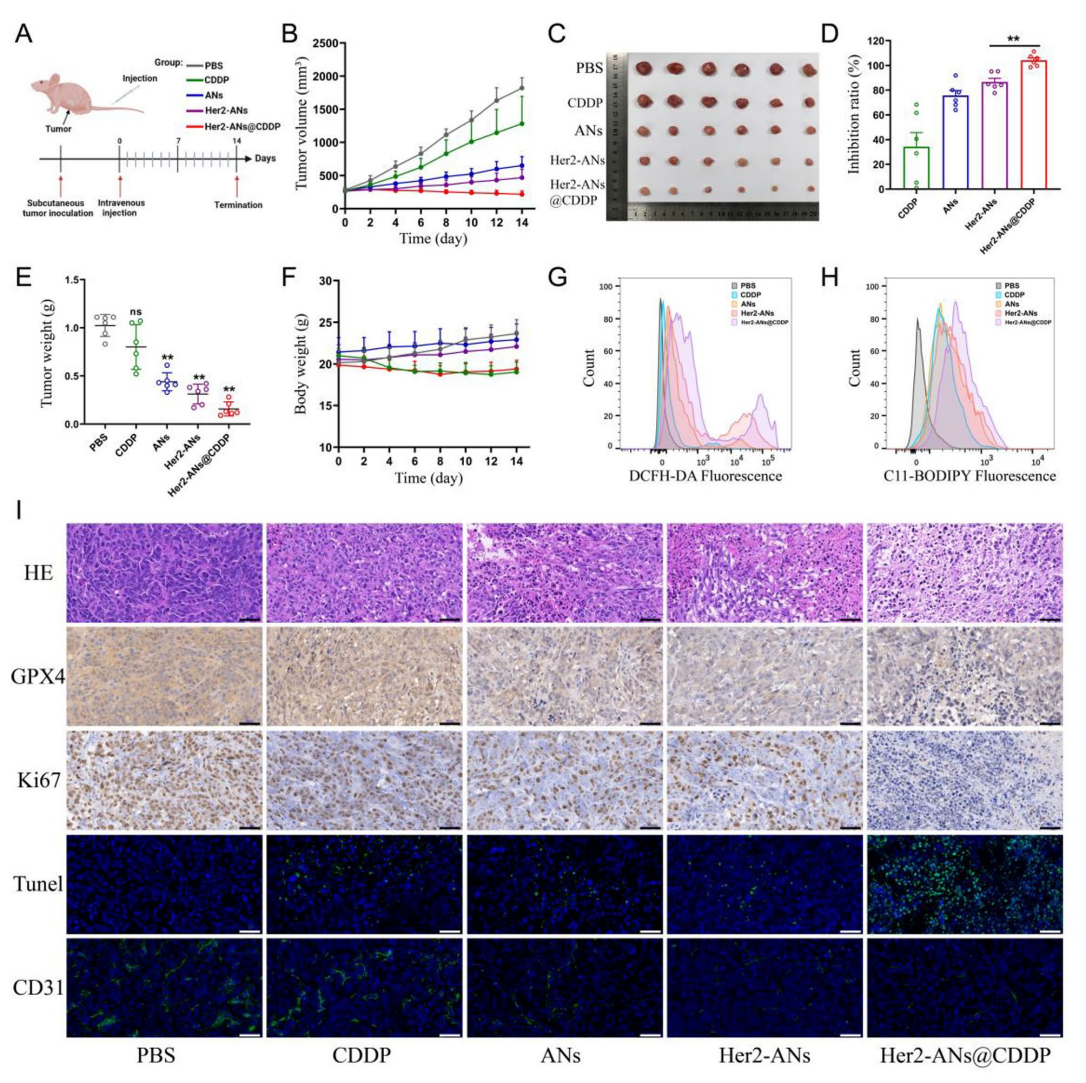
Therapeutic efficacy of Her2-ANs in vivo. **(A)** In vivo tumor treatment schedule. **(B)** Tumor growth curves of different groups. **(C)** Tumor photos after various treatments. **(D)** Tumor inhibition rates of different groups. (Values are presented as means ± sd, n = 6, *P < 0.05, **P < 0.01.) **(E)** The final weight of tumor tissues extracted from mice after different treatments. (Values are presented as means ± sd, n = 6, *P < 0.05, **P < 0.01.) **(F)** Body weight changes in mice within 2 weeks after different treatments. **(G, H)** Flow cytometry analysis of the production of ROS **(G)** and lipid peroxide **(H)** in the tumor of different groups. **(I)** HE, immunohistochemical and immunofluorescence analysis of tumor slices obtained from different groups. Scale bar: 50 μm

Because of the excellent in vitro antitumor activity of ANs, we further evaluated the in vivo antitumor effects of ANs. A monoclonal formation assay showed that Her2-ANs and Her2-ANs@CDDP could completely inhibit the formation of tumor cell colonies in vitro (Additional file 1: Fig. S12). To investigate the tumor-homing capacity of target-modified ANs, the distribution of the nanosheets in vivo was analyzed by ICP-MS. The results showed a significantly reduced accumulation of Her2-ANs in the mononuclear phagocytic system of the liver, however, there was a significant increase in the content of ANs within the tumor (Additional file 1: Fig. S13). During 14 days of treatment, the tumor volume of mice in the control and cisplatin groups increased rapidly by more than 1000 mm^3^, while the tumor volumes of mice in the other groups showed a significant suppression (Fig. 6B). Among all groups, the Her2-ANs@CDDP group had the highest tumor inhibition rate, which was attributed to the antitumor effect of ferroptosis induced by GSH depletion in combination with the targeted delivery of cisplatin (Fig. 6D).

To explore the mechanism by which ANs induce tumor cell death in vivo, we measured the levels of ROS and lipid peroxides in fresh tumor tissues. As shown in Fig. 6G, H, the levels of ROS and lipid peroxides were significantly elevated in tumor tissues of the Her2-ANs@CDDP group. In addition, immunohistochemical staining showed significantly lower expression levels of GPX4 in tumor tissues of the Her2-ANs@CDDP group compared with the cisplatin group (Fig. 6I). These results suggest that ANs can induce ferroptosis of tumor cells in vivo by inhibiting the expression of GPX4, resulting in suppression of tumor tissue growth. In addition, pathological analyses were performed on sections of tumor tissues to comprehensively assess the antitumor effects of Her2-ANs@CDDP. Hematoxylin and eosin (HE) staining showed large necrotic areas mixed with cellular debris in the tumor tissue, indicating that Her2-ANs@CDDP effectively induces tumor tissue damage (Fig. 6I). These findings were further confirmed by TUNEL staining, which showed that the tumor tissue was filled with apoptotic cells. The proliferation status of tumor cells was assessed by the expression of Ki67 protein. There was almost complete absence of Ki67 expression in the tumor tissues of the Her2-ANs@CDDP group. CD31 staining showed a significant reduction of microvessels in tumor tissue of the Her2-ANs@CDDP group, which may be attributed to significant inhibition of tumor cell activity resulting in reduced angiogenesis [49]. Therefore, the synergistic effect of Her2-ANs@CDDP through induction of ferroptosis and apoptosis achieved a comprehensive inhibition of OS progression.

### Biosafety assessment of ANs

In addition to assessing the in vivo antitumor effects of the nanosheets, attention was also focused on the safety and reliability of the nanosheets after intravenous injection. The results of a hemolysis assay showed that ANs did not cause erythrocyte rupture at multiple concentration gradients, suggesting that ANs can be administered by intravenous injection (Additional file 1: Fig. S17). Compared with the cisplatin group, mice in the Her2-ANs@CDDP group had only a slight decrease in body weight, which was gradually restored over time (Fig. 6F). There were no observable necrosis, inflammatory lesions, or histological abnormalities in HE sections of the major organs of the mice (Additional file 1: Fig. S16). In addition, the hematological and blood biochemical indicators of the mice fluctuated within the normal range after treatment (Additional file 1: Fig. S14, Fig. S15). In conclusion, cisplatin-loaded ANs have excellent biosafety in vivo and are expected to be applied to OS treatment through clinical translation in the future.

## Conclusions

We prepared Her2-ANs@CDDP through liquid exfoliation techniques and targeted modifications. As Her2 is overexpressed in osteosarcoma, it can be used as a target for osteosarcoma therapy. Unlike previously reported metal nanomaterials that induce ferroptosis by the Fenton reaction, ANs can be used as a new ferroptosis inducer by depleting intracellular GSH and can significantly inhibit the activity of drug-resistant cells. Her2-ANs@CDDP promotes apoptosis and ferroptosis through a reciprocal cascade of reactions between cisplatin and carrier, respectively. The ROS produced by cisplatin synergistically promotes ANs-induced ferroptosis through increased levels of oxidative stress. ANs restore the sensitivity of drug-resistant cells to cisplatin by increasing the accumulation of intracellular cisplatin. Multifunctional nanocarriers can be prepared simply and exert synergistic effects that overcome drug resistance. They show great potential for applications in the clinical treatment of chemotherapy-insensitive osteosarcoma and expand the potential uses of arsenic in the treatment of solid tumors.

## Electronic supplementary material

Below is the link to the electronic supplementary material.

Additional file 1: Figure S1. Single-cell RNA sequencing analysis for Her2 expression in human OS. Figure S2. U2OS and 143B cells were sorted by sorting flow cytometry into Her2(+) and Her2(-) subpopulations. Figure S3. Characterization of the raw arsenic powder. Figure S4. Characterization of the ANs and Her2-ANs. Figure S5. TEM images of Her2-ANs at different pH values and different GSH concentrations. Figure S6.In vitro GSH-depleting effects of Her2-ANs. Figure S7. (A, B) Flow cytometry analysis of cell death in U2OS-Her2(+) cells treated with various treatments. (C) Relative viabilities of U2OS-Her2(+), U2OS-Her2(-) and AML-12 cells after incubation with Her2-ANs at different concentrations. Figure S8. (A) Intracellular uptake of ANs, Her2-ANs and raw arsenic powder in the U2OS-Her2(+) cells was measured by ICP-MS. (B) The viability of U2OS-Her2(+) cells treated with Her2-ANs was rescued by various inhibitors. Figure S9. Detection of intracellular ROS and lipid peroxidation. Figure S10. Statistical analysis of Western blot results for GPX4 expression in U2OS-Her2(+) cells after treatment with Her2-ANs at different concentrations and times. Figure S11. Cytotoxicity of CDDP against the U2OS and U2OS/CDDP cells was determined by CCK-8 assay. Figure S12. The proliferation ability of the 143B-Her2(+) cells with different treatments was detected by colony formation assay. Figure S13. The accumulation of As ions in the major organs or tumors of tumor-bearing nude mice evaluated by ICP-MS at 24 h. Figure S14. Hematological parameters of tumor-bearing mice after intravenous injection of PBS, CDDP, ANs, Her2-ANs and Her2-ANs@CDDP for 2 weeks. Figure S15. Blood biochemical indicators of tumor-bearing mice after intravenous injection of PBS, CDDP, ANs, Her2-ANs and Her2-ANs@CDDP for 2 weeks. Figure S16. Histopathological examination of the major organs of tumor-bearing mice after intravenous injection of PBS, CDDP, ANs, Her2-ANs and Her2-ANs@CDDP for 2 weeks. Figure S17. (A) The analysis of the hemolysis rate. (B) Pictures of Her2-ANs incubated with blood for 8 h. Table S1. Details of the osteosarcoma samples used for single-cell sequencing. Table S2. Details of the osteosarcoma samples used for immunohistochemistry.

## Data Availability

All data generated or analyzed during this study are included in this article.

## References

[1] He P, Xu S, Guo Z, Yuan P, Liu Y, Chen Y, Zhang T, Que Y, Hu Y (2022). Pharmacodynamics and pharmacokinetics of PLGA-based doxorubicin-loaded implants for tumor therapy. Drug Delivery.

[2] Gill J, Gorlick R. Advancing therapy for osteosarcoma. Nat Rev Clin Oncol 2021. 10.1038/s41571-021-00519-8 34131316

[3] Song L, Duan P, Gan Y, Li P, Zhao C, Xu J, Zhang Z, Zhou Q (2017). Silencing LPAATbeta inhibits tumor growth of cisplatin-resistant human osteosarcoma in vivo and in vitro. Int J Oncol.

[4] Fu J, Li T, Yang Y, Jiang L, Wang W, Fu L, Zhu Y, Hao Y (2021). Activatable nanomedicine for overcoming hypoxia-induced resistance to chemotherapy and inhibiting tumor growth by inducing collaborative apoptosis and ferroptosis in solid tumors. Biomaterials.

[5] Ma MZ, Chen G, Wang P, Lu WH, Zhu CF, Song M, Yang J, Wen S, Xu RH, Hu Y, Huang P (2015). Xc- inhibitor sulfasalazine sensitizes colorectal cancer to cisplatin by a GSH-dependent mechanism. Cancer Lett.

[6] Zhang C, Wang P, Zhang YN, Lu P, Huang X, Wang Y, Ran L, Xin H, Xu X, Gao W (2023). Biodegradable nanoplatform upregulates tumor microenvironment acidity for enhanced cancer therapy via synergistic induction of apoptosis, ferroptosis, and anti-angiogenesis. J Nanobiotechnol.

[7] Hassannia B, Vandenabeele P, Vanden Berghe T (2019). Targeting ferroptosis to Iron Out Cancer. Cancer Cell.

[8] Zhang P, Fu J, Hu J, You Q, Yao X, Hua D, Yin J, Mao Y (2023). Evoking and enhancing ferroptosis of cancer stem cells by a liver-targeted and metal-organic framework-based drug delivery system inhibits the growth and lung metastasis of hepatocellular carcinoma. Chem Eng J.

[9] Zanganeh S, Hutter G, Spitler R, Lenkov O, Mahmoudi M, Shaw A, Pajarinen JS, Nejadnik H, Goodman S, Moseley M (2016). Iron oxide nanoparticles inhibit tumour growth by inducing pro-inflammatory macrophage polarization in tumour tissues. Nat Nanotechnol.

[10] Zheng DW, Lei Q, Zhu JY, Fan JX, Li CX, Li C, Xu Z, Cheng SX, Zhang XZ (2017). Switching apoptosis to ferroptosis: Metal-Organic Network for High-Efficiency Anticancer Therapy. Nano Lett.

[11] Dixon SJ, Stockwell BR (2014). The role of iron and reactive oxygen species in cell death. Nat Chem Biol.

[12] Zhang P, Cui Y, Wang J, Cheng J, Zhu L, Liu C, Yue S, Pang R, Guan J, Xie B (2023). Dual-stimuli responsive smart nanoprobe for precise diagnosis and synergistic multi-modalities therapy of superficial squamous cell carcinoma. J Nanobiotechnol.

[13] Tang H, Li C, Zhang Y, Zheng H, Cheng Y, Zhu J, Chen X, Zhu Z, Piao JG, Li F (2020). Targeted Manganese doped silica nano GSH-cleaner for treatment of Liver Cancer by destroying the intracellular redox homeostasis. Theranostics.

[14] Zhao D, Huang X, Tian Y, Zou J, Wang F, Chen X (2023). Fluorescence Imaging-Incorporated Transcriptome Study of glutathione depletion-enhanced ferroptosis therapy via Targeting Gold Nanoclusters. ACS Appl Mater Interfaces.

[15] Wang X, Hu Y, Mo J, Zhang J, Wang Z, Wei W, Li H, Xu Y, Ma J, Zhao J (2020). Arsenene: a potential therapeutic Agent for Acute promyelocytic leukaemia cells by acting on Nuclear Proteins. Angew Chem Int Ed Engl.

[16] Miller W, Schipper H, Lee J, Singer J. Waxman SJCr: mechanisms of action of arsenic trioxide. 2002, 62:3893–903. 12124315

[17] Snow ET (1992). Metal carcinogenesis: mechanistic implications. Pharmacol Ther.

[18] Chen S, Wu JL, Liang Y, Tang YG, Song HX, Wu LL, Xing YF, Yan N, Li YT, Wang ZY (2021). Arsenic Trioxide rescues structural p53 mutations through a cryptic allosteric site. Cancer Cell.

[19] Jiang Y, Cheng J, Yang C, Hu Y, Li J, Han Y, Zang Y, Li X (2017). An ultrasensitive fluorogenic probe for revealing the role of glutathione in chemotherapy resistance. Chem Sci.

[20] Mansur AAP, Mansur HS, Leonel AG, Carvalho IC, Lage MCG, Carvalho SM, Krambrock K, Lobato ZIP (2020). Supramolecular magnetonanohybrids for multimodal targeted therapy of triple-negative breast cancer cells. J Mater Chem B.

[21] Fathi P, Roslend A, Alafeef M, Moitra P, Dighe K, Esch MB, Pan D (2022). In situ Surface-Directed Assembly of 2D metal nanoplatelets for drug-free treatment of antibiotic-resistant Bacteria. Adv Healthc Mater.

[22] Wang Z, Liu G, Chen W, Zhang L, Qi Z, Bai G, Fan Y, Liu C, Xiao C, Li W (2023). Contribution of surface plasmonic resonance to enhanced photocatalytic antibacterial performance of graphene-based two-dimensional heterojunction. Chem Eng J.

[23] Matos AI, Carreira B, Peres C, Moura LIF, Conniot J, Fourniols T, Scomparin A, Martinez-Barriocanal A, Arango D, Conde JP (2019). Nanotechnology is an important strategy for combinational innovative chemo-immunotherapies against colorectal cancer. J Controlled Release.

[24] Modi S, Park H, Murthy RK, Iwata H, Tamura K, Tsurutani J, Moreno-Aspitia A, Doi T, Sagara Y, Redfern C (2020). Antitumor Activity and Safety of Trastuzumab Deruxtecan in patients with HER2-Low-expressing advanced breast Cancer: results from a phase ib study. J Clin Oncol.

[25] Ahmed N, Brawley VS, Hegde M, Robertson C, Ghazi A, Gerken C, Liu E, Dakhova O, Ashoori A, Corder A (2015). Human epidermal growth factor receptor 2 (HER2) -Specific chimeric Antigen receptor-modified T cells for the Immunotherapy of HER2-Positive sarcoma. J Clin Oncol.

[26] Ebb D, Meyers P, Grier H, Bernstein M, Gorlick R, Lipshultz SE, Krailo M, Devidas M, Barkauskas DA, Siegal GP (2012). Phase II trial of trastuzumab in combination with cytotoxic chemotherapy for treatment of metastatic osteosarcoma with human epidermal growth factor receptor 2 overexpression: a report from the children’s oncology group. J Clin Oncol.

[27] Camacho LH, Soignet SL, Chanel S, Ho R, Heller G, Scheinberg DA, Ellison R, Warrell RP (2000). Leukocytosis and the retinoic acid syndrome in patients with acute promyelocytic leukemia treated with arsenic trioxide. J Clin Oncol.

[28] Chang YY, Kuo TC, Hsu CH, Hou DR, Kao YH, Huang RN (2012). Characterization of the role of protein-cysteine residues in the binding with sodium arsenite. Arch Toxicol.

[29] Ito K, Bernardi R, Morotti A, Matsuoka S, Saglio G, Ikeda Y, Rosenblatt J, Avigan DE, Teruya-Feldstein J, Pandolfi PP (2008). PML targeting eradicates quiescent leukaemia-initiating cells. Nature.

[30] Lu N, Tian Y, Tian W, Huang P, Liu Y, Tang Y, Wang C, Wang S, Su Y, Zhang Y (2016). Smart Cancer Cell Targeting Imaging and Drug Delivery System by systematically Engineering Periodic Mesoporous Organosilica Nanoparticles. ACS Appl Mater Interfaces.

[31] Yang M, Cheng K, Qi S, Liu H, Jiang Y, Jiang H, Li J, Chen K, Zhang H, Cheng Z (2013). Affibody modified and radiolabeled gold-iron oxide hetero-nanostructures for tumor PET, optical and MR imaging. Biomaterials.

[32] Ma P, Xiao H, Yu C, Liu J, Cheng Z, Song H, Zhang X, Li C, Wang J, Gu Z, Lin J (2017). Enhanced cisplatin chemotherapy by Iron oxide nanocarrier-mediated generation of highly toxic reactive oxygen species. Nano Lett.

[33] Ling X, Chen X, Riddell IA, Tao W, Wang J, Hollett G, Lippard SJ, Farokhzad OC, Shi J, Wu J (2018). Glutathione-scavenging poly(disulfide amide) nanoparticles for the effective delivery of pt(IV) prodrugs and reversal of Cisplatin Resistance. Nano Lett.

[34] Ferraro G, Massai L, Messori L, Merlino A (2015). Cisplatin binding to human serum albumin: a structural study. Chem Commun (Camb).

[35] Messori L, Marzo T, Michelucci E, Russo Krauss I, Navarro-Ranninger C, Quiroga AG, Merlino A (2014). Interactions between anticancer trans-platinum compounds and proteins: crystal structures and ESI-MS spectra of two protein adducts of trans-(dimethylamino)(methylamino)dichloridoplatinum(II). Inorg Chem.

[36] Huliciak M, Reinhard L, Laursen M, Fedosova N, Nissen P, Kubala M (2014). Crystals of na(+)/K(+)-ATPase with bound cisplatin. Biochem Pharmacol.

[37] Cheng JJ, Zhu Y, Xing X, Xiao JM, Chen H, Zhang HW, Wang D, Zhang YY, Zhang GL, Wu ZY, Liu YZ (2021). Manganese-deposited iron oxide promotes tumor-responsive ferroptosis that synergizes the apoptosis of cisplatin. Theranostics.

[38] Zhang C, Liu X, Jin S, Chen Y, Guo R (2022). Ferroptosis in cancer therapy: a novel approach to reversing drug resistance. Mol Cancer.

[39] Circu ML, Aw TY (2010). Reactive oxygen species, cellular redox systems, and apoptosis. Free Radic Biol Med.

[40] Rybak LP, Mukherjea D, Jajoo S, Ramkumar V (2009). Cisplatin ototoxicity and protection: clinical and experimental studies. Tohoku J Exp Med.

[41] Johnstone TC, Suntharalingam K, Lippard SJ (2016). The Next Generation of Platinum Drugs: targeted pt(II) agents, nanoparticle delivery, and pt(IV) Prodrugs. Chem Rev.

[42] Wang D, Zhang G, Zhou L, Wang M, Cai D, Wu Z (2017). Synthesis of a multifunctional graphene oxide-based magnetic nanocomposite for efficient removal of cr(VI). Langmuir.

[43] Stockwell BR (2019). A powerful cell-protection system prevents cell death by ferroptosis. Nature.

[44] Dizdaroglu M, Jaruga P (2012). Mechanisms of free radical-induced damage to DNA. Free Radic Res.

[45] Zhong B, Shi D, Wu F, Wang S, Hu H, Cheng C, Qing X, Huang X, Luo X, Zhang Z, Shao Z (2019). Dynasore suppresses cell proliferation, migration, and invasion and enhances the antitumor capacity of cisplatin via STAT3 pathway in osteosarcoma. Cell Death Dis.

[46] Dixon SJ, Lemberg KM, Lamprecht MR, Skouta R, Zaitsev EM, Gleason CE, Patel DN, Bauer AJ, Cantley AM, Yang WS (2012). Ferroptosis: an iron-dependent form of nonapoptotic cell death. Cell.

[47] Galluzzi L, Senovilla L, Vitale I, Michels J, Martins I, Kepp O, Castedo M, Kroemer G (2012). Molecular mechanisms of cisplatin resistance. Oncogene.

[48] Wu WJ, Zhang Y, Zeng ZL, Li XB, Hu KS, Luo HY, Yang J, Huang P, Xu RH (2013). beta-phenylethyl isothiocyanate reverses platinum resistance by a GSH-dependent mechanism in cancer cells with epithelial-mesenchymal transition phenotype. Biochem Pharmacol.

[49] Jiang W, Luo X, Wei L, Yuan S, Cai J, Jiang X, Hu Y. The sustainability of Energy Conversion Inhibition for Tumor Ferroptosis Therapy and Chemotherapy. Small 2021:e2102695. 10.1002/smll.202102695 34350694

