# Supplementary material for: Anti-Her2 affibody-decorated arsenene nanosheets induce ferroptosis through depleting intracellular GSH to overcome cisplatin resistance
Source: J Nanobiotechnology. 2023 Jun 27;21:203. doi: 10.1186/s12951-023-01963-7 (PMC10294336; doi:10.1186/s12951-023-01963-7)
Supplement: Supplementary file 1 — Additional file 1: Figure S1. Single-cell RNA sequencing analysis for Her2 expression in human OS. Figure S2. U2OS and 143B cells were sorted by sorting flow cytometry into Her2(+) and Her2(-) subpopulations. Figure S3. Characterization of the raw arsenic powder. Figure S4. Characterization of the ANs and Her2-ANs. Figure S5. TEM images of Her2-ANs at different pH values and different GSH concentrations. Figure S6.In vitro GSH-depleting effects of Her2-ANs. Figure S7. (A, B) Flow cytometry analysis of cell death in U2OS-Her2(+) cells treated with various treatments. (C) Relative viabilities of U2OS-Her2(+), U2OS-Her2(-) and AML-12 cells after incubation with Her2-ANs at different concentrations. Figure S8. (A) Intracellular uptake of ANs, Her2-ANs and raw arsenic powder in the U2OS-Her2(+) cells was measured by ICP-MS. (B) The viability of U2OS-Her2(+) cells treated with Her2-ANs was rescued by various inhibitors. Figure S9. Detection of intracellular ROS and lipid peroxidation. Figure S10. Statistical analysis of Western blot results for GPX4 expression in U2OS-Her2(+) cells after treatment with Her2-ANs at different concentrations and times. Figure S11. Cytotoxicity of CDDP against the U2OS and U2OS/CDDP cells was determined by CCK-8 assay. Figure S12. The proliferation ability of the 143B-Her2(+) cells with different treatments was detected by colony formation assay. Figure S13. The accumulation of As ions in the major organs or tumors of tumor-bearing nude mice evaluated by ICP-MS at 24 h. Figure S14. Hematological parameters of tumor-bearing mice after intravenous injection of PBS, CDDP, ANs, Her2-ANs and Her2-ANs@CDDP for 2 weeks. Figure S15. Blood biochemical indicators of tumor-bearing mice after intravenous injection of PBS, CDDP, ANs, Her2-ANs and Her2-ANs@CDDP for 2 weeks. Figure S16. Histopathological examination of the major organs of tumor-bearing mice after intravenous injection of PBS, CDDP, ANs, Her2-ANs and Her2-ANs@CDDP for 2 weeks. Figure S17. (A [file 12951_2023_1963_MOESM1_ESM.docx]

Additional file 1

**Anti-Her2 Affibody-decorated Arsenene Nanosheets Induce Ferroptosis through Depleting Intracellular GSH to Overcome Cisplatin Resistance**

Peng He^1, †^, Shenglin Xu^1, †^, Zhaohua Miao^2^, Yukang Que^1^, Yu Chen^3^, Sheng Li^1^, Qiming Ma^1^, Rui Yang^1^, Wei Wei^4, *^, Zhengbao Zha^2, *^ and Yong Hu^1, *^

^1^ *Department of Orthopedics, The First Affiliated Hospital of Anhui Medical University, Hefei 230022, Anhui, China*

^2^ *School of Food and Biological Engineering, Hefei University of Technology, Hefei 230009, Anhui, China*

^3^ *Department of Pharmacy, Anqing Medical College, Anqing 246052, Anhui, China*

^4^ *Institute of Clinical Pharmacology, Anhui Medical University, Hefei 230032, Anhui, China*

**Correspondence:* wwei@ahmu.edu.cn; zbzha@hfut.edu.cn; hy.in163@163.com.

^†^ Peng He and Shenglin Xu contributed equally.

**Experimental Section**

**Materials**

Raw arsenic powder and 5(6)-carboxyfluorescein N-hydroxysuccinimide ester were purchased from Sigma (St. Louis, MO, USA). Cisplatin and bovine serum albumin (BSA) were ordered from Aladdin (Shanghai, China). SH-PEG-NH2 was obtained from Shanghai Yare Biotech, Inc. (Shanghai, China). CCK-8 was purchased from Biosharp Life Sciences (Hefei, China). Glutathione (GSH), Ferrostatin-1 (Fer-1), Acetylcysteine (NAC), Z-VAD-FMK, Deferoxamine mesylate (DFOM) and 5,5'-dithiobis (2-nitrobenzoic acid) (DTNB) were ordered from MCE (Shanghai, China). 2′,7′-dichlorofluorescin diacetate (DCFH-DA) was obtained from Beyotime (Shanghai, China). BODIPY 581/591 C11 was purchased from Thermo Fisher (Waltham, MA, USA). Glutaraldehyde and hexamethyl disilylamine were purchased from Macklin (Shanghai, China). Hoechst 33342 was ordered from APExBIO (Houston, TX, USA). 4 ′ ,6-diamidino-2-phenylindole (DAPI) was obtained from Solarbio (Beijing, China). Fetal bovine serum (FBS) was purchased from Biological Industries® (Kibbutz Beit Haemek, Israel).

**Single-cell RNA sequencing analysis for Her2 expression in OS** **derived from patients**

Five patients hospitalized in the First Affiliated Hospital of Anhui Medical University from June 2021 to July 2022 were enrolled in this study, which was approved by the Ethics Committee of the First Affiliated Hospital of Anhui Medical University (PJ2022-12-52). Five patients (two male and three female, age range from 7 to 55 years) were diagnosed with OS based on the final pathological results. Samples were subjected to RNA sequencing after tissue dissociation and library preparation.

**Immunohistochemical analysis for Her2 expression in OS derived from patients**

Eight patients hospitalized in the First Affiliated Hospital of Anhui Medical University from March 2021 to August 2022 were enrolled in this study, which was approved by the Ethics Committee of the First Affiliated Hospital of Anhui Medical University (PJ2022-12-52). Eight patients (four male and four female, age range from 7 to 55 years) were diagnosed with OS based on the final pathological results. The expression of Her2 in osteosarcoma was analyzed by immunohistochemistry. Paraffin sections of the tumor were immunostained with an antibody against Her2 (ER0106, 1:200, HuaBio, China).

**Western blotting analysis for Her2 expression in OS cell lines**

To analyze the expression of Her2 protein in U2OS and 143B cells, total protein was collected after lysing the cells. The proteins were then separated by SDS-PAGE gel electrophoresis and transferred to polyvinylidene difluoride (PVDF) membranes. PVDF membranes were blocked with 5% skim milk and then incubated with primary antibodies for Her2 (ER0106, 1:500, HuaBio, China) and GAPDH (T0004, 1:1000, Affinity, China) overnight.

**The expression of Her2 in human-derived OS cell lines was analyzed by flow cytometry**

5 ×10^5^ cells were incubated with PE-Her2 antibody (324405, 1 μL, Biolegend, USA) for 30 minutes. The expression of Her2 in osteosarcoma cells was tested using a flow cytometer (FC500, Beckman). According to the above method, osteosarcoma cells were sorted by sorting flow cytometry (FACSAria II, BD Biosciences, USA) into Her2(+) and Her2(-) subpopulations for subsequent experiments.

**Preparation of ANs**

Raw arsenic powder (100 mg) was ground to a fine powder using a grinding bowl. The arsenic powder was added to a 50 mL centrifuge tube containing 10 mL of anhydrous ethanol and 20 mL of ultrapure water. The mixture was treated with an ultrasound probe at 500 W in a discontinuous mode (work time 3 seconds, interval time 3 seconds) for 5 hours under an ice bath. The ultrasound-treated solution was placed in an ultrasonic bath and continued to be sonicated at 300 W for 5 hours under an ice bath. The resulting arsenic solution was centrifuged at 3000 rpm for 15 minutes. The supernatant was collected and centrifuged at 10,000 rpm for 15 minutes to obtain arsenene nanosheets (ANs). ANs were washed three times with ultrapure water, resuspended using deoxygenated water, and stored at 4℃ in the dark.

**Preparation of Her2-ANs**

To increase the tumor homing capacity of the nanosheets, 50 μg of Anti-Her2 Affibody (ab31889, Abcam, UK) and 200 μg of ANs were added to 1.5 mL of ultrapure water and stirred overnight. The mixture was centrifuged at 12,000 rpm for 20 minutes to obtain Her2-ANs. Her2-ANs were washed three times with ultrapure water, resuspended using deoxygenated water and stored at 4℃ in the dark.

**Drug loading and release**

Before cisplatin loading, the surface of the material is modified using BSA. 1.5 mg of BSA and 200 μg of Her2-ANs were added to 1.5 mL of ultrapure water and stirred for 2 hours. Cisplatin was added to this solution so that the final concentration of cisplatin was 0.125, 0.25, 0.5, 0.75, or 1 mg/mL. The mixtures were stirred for 20 hours and then centrifuged at 12000 rpm for 15 minutes to pellet the Her2-ANs@CDDP. The Her2-ANs@CDDP was washed three times with ultrapure water and then resuspended using deoxygenated water and stored at 4℃ in the dark. Her2-ANs@CDDP samples obtained from different cisplatin concentrations were treated with aqua regia and then the content of loaded Pt was determined by an Inductively Coupled Plasma Optic Emission Spectrometer (ICP-OES, Opmita 7300 DV, Perkin Elmer, USA).

To evaluate the drug release properties of Her2-ANs@CDDP *in vitro*, 0.5 mL of Her2-ANs@CDDP was sealed in a dialysis bag and placed in a centrifuge tube containing 9.5 mL of different release media (pH=7.4 or 5.0; GSH=0 or 5 mM). The centrifuge tubes were then placed on a shaker at 37°C and 100 μL of the release solution was collected at a predetermined time point. The amount of Pt in the release solution was determined by Liquid Chromatograph Inductively Coupled Plasma Mass Spectrometry (ICP-MS, NexION 5000, Perkin Elmer, USA).

**Preparation of FITC-labeled ANs and Her2-ANs**

1 mg of SH-PEG-NH2 and 200 μg of ANs (Her2-ANs) were added to 2 mL of ultrapure water and stirred overnight. The mixture was washed three times with ultrapure water to remove the unreacted SH-PEG-NH2. Then 1 mg of 5(6)-carboxyfluorescein N-hydroxysuccinimide ester (FITC) was added to the above solution and stirred at pH 8.0 for 24 hours. The above mixture was centrifuged at 12,000 rpm for 20 minutes to obtain the precipitate (ANs-FITC or Her2-ANs-FITC). The precipitate was washed three times with ultrapure water, resuspended using deoxygenated water and stored at 4℃ in the dark.

**Characterization**

Transmission electron microscopy images of nanomaterials were taken by transmission electron microscopy (TEM, Talos L120C, Thermo Scientific, USA). High-resolution transmission electron microscopy images were taken by field emission transmission electron microscopy (HRTEM, JEM-2100F, JEOL, Japan). Topographic images and height profiles of the material were collected by atomic force microscopy (AFM, Dimension Icon, Bruker, Germany). The infrared features of ANs and Her2-ANs were measured by the Fourier transform infrared spectrometer (FT-IR, Nicolet 8700, Thermo Scientific, USA). The Raman spectra were obtained on a confocal laser Raman spectroscope (LabRAM HR Evolution, HORIBA JOBIN YVON, France) using a laser with a 532 nm wavelength. The distributions and proportions of As, Pt, O and S elements in the nanosheets were obtained by EDS elemental mapping. The UV-Visible absorption spectra were measured by UV spectrophotometer (Q5000, Quawell, USA). The size distribution and zeta potential of the nanosheets were evaluated using a Zetasizer (Nano ZS90, Malvern, UK).

**GSH-depleting effects of Her2-ANs**

Ellman's reagent was used to measure GSH levels by the DTNB assay. Her2-ANs were added to the GSH solution (3 mM) and incubated at 37°C for different time intervals (0, 1, 2, 4, 8, 12, and 24 hours). The samples were centrifuged at 10000 rpm for 15 minutes and each supernatant was collected to evaluate GSH levels. The supernatant and DTNB solution (4 mM) were co-incubated for 5 minutes and the absorbance was measured at 412 nm using a microplate reader (Infinite M1000 PRO, TECAN, Switzerland).

**Establishment of cisplatin-resistant cell lines**

Cisplatin-resistant U2OS/CDDP cells were established by prolonged exposure of parental cells to progressively increasing concentrations of cisplatin. The progressively increasing drug concentrations were 6.4 ng/mL, 32 ng/mL, 160 ng/mL, 800 ng/mL and 4000 ng/mL, respectively. To maintain cisplatin resistance, the established U2OS/CDDP cells need to be continuously cultured at their screened drug concentration.

**Targeted uptake evaluation by Flow Cytometry**

U2OS-Her2(+) cells (6×10^4^ cells/well) were seeded into 24-well plates and cultured overnight. The cells were treated with culture medium containing 5 μg/mL nanosheets (ANs-FITC or Her2-ANs-FITC) and cultured for different time intervals. Then, the cells were washed three times with PBS and collected into flow tubes. Finally, the fluorescence signal at each time point was detected by flow cytometry (FC500, Beckman).

**Targeted uptake evaluation by Confocal Laser Scanning Microscopy**

U2OS-Her2(+) cells (6×10^4^ cells/well) were seeded into 24-well plates containing round coverslip and cultured overnight. The cells were treated with culture medium containing 10 μg/mL nanosheets (ANs-FITC or Her2-ANs-FITC) and cultured for different time intervals. Subsequently, the nuclei were stained with Hoechst 33342 for 15 minutes. Finally, the fluorescence image at each time point was observed by confocal laser scanning microscopy (DFC 7000T, Leica, Germany).

**Targeted uptake evaluation by ICP-MS**

U2OS-Her2(+) cells (3×10^5^ cells/well) were seeded into 6-well plates and cultured overnight. The cells were treated with culture medium containing 5 μg/mL nanomaterial (AS or ANs or Her2-ANs) and cultured for 4 hours. Then, the cells were washed three times with PBS and collected into centrifuge tubes. The cells in the centrifuge tubes were frozen overnight at -80°C and then lysed with aqua regia at 95°C for 3 hours. The intracellular As content was determined by ICP-MS (NexION 5000, Perkin Elmer, USA).

**In vitro cell viability assays**

The cytotoxic effects of cisplatin, nanosheets and cisplatin-loaded nanosheets on U2OS-Her2(+), U2OS-Her2(-), U2OS/CDDP, 143B-Her2(+), 143B-Her2(-) and AML-12 cells were detected by CCK-8 assay. The cells (9×10^3^ cells/well) were seeded into 96-well plates and cultured overnight. The culture medium was replaced with 100 μL of fresh medium containing free CDDP, ANs, Her2-ANs, ANs@CDDP, or Her2-ANs@CDDP (CDDP: 2 μg/mL, As ions: 10 μg/mL) and the cells were cultured for 20 hours. Finally, 10 μL of CCK-8 was added to the well. The absorbance of each well at 450 nm was measured using a microplate reader (Infinite M1000 PRO, TECAN, Switzerland).

**In vitro cell viability assay after treatment with various inhibitors**

U2OS-Her2(+) cells (9×10^3^ cells/well) were seeded into 96-well plates and cultured overnight. After GSH (8 mM), ferroptosis inhibitor (Fer-1; 10 μM), NAC (2 mM), DFOM (100 μM) and apoptosis inhibitor (Z-VAD-FMK; 20 μM) were added to 96-well plates for 1 hour, the cells were treated with 10 μg/mL of Her2-ANs. After 20 hours, the cells were subjected to a CCK-8 assay.

**Intracellular GSH content**

U2OS-Her2(+) cells (6×10^4^ cells/well) were seeded into 24-well plates and cultured overnight. The cells were treated with culture medium containing different concentrations of Her2-ANs and incubated for 8 hours. Intracellular glutathione content was measured using the Glutathione Assay Kit (S0053, Beyotime, China) according to the manufacturer's instructions. The absorbance of each well at 412 nm was measured using a microplate reader (Infinite M1000 PRO, TECAN, Switzerland). To compare the GSH content in U2OS and U2OS/CDDP cells, 2×10^5^ cells were collected into a centrifuge tube and detected according to the above method.

**Intracellular GPX4 activity assay**

U2OS-Her2(+) cells (6×10^4^ cells/well) were seeded into 24-well plates and cultured overnight. The cells were treated with culture medium containing different concentrations of Her2-ANs and incubated for 8 hours. Intracellular GPX4 activity was detected using the Glutathione Peroxidase Assay Kit (S0056, Beyotime, China) according to the manufacturer's instructions. The absorbance of each well at 340 nm was measured using a microplate reader (Infinite M1000 PRO, TECAN, Switzerland).

**Western blot analysis**

U2OS-Her2(+) cells (3×10^5^ cells/well) were seeded into 6-well plates and cultured overnight. Her2-ANs were added and incubated at different concentrations for 8 hours or at 40 μg/mL for 0, 2, 4, and 8 hours. Total cellular proteins were collected and analyzed by western blotting as described above. The proteins on the PVDF membrane were incubated overnight with primary antibodies of GPX4 (R24461, 1:500, ZenBio, China).

**Detection of intracellular ROS and lipid peroxidation**

U2OS-Her2(+) or U2OS/CDDP cells (6×10^4^ cells/well) were seeded into 24-well plates and cultured overnight. The cells were treated with media containing different concentrations of Her2-ANs (0, 10, 20 or 40 μg/mL) or nanosheets loaded with cisplatin (PBS, CDDP, Her2-ANs or Her2-ANS@CDDP) and incubated for 8 hours. To detect by flow cytometry the ROS that were generated or lipid peroxidation, cells were stained with a ROS-sensitive probe (DCFH-DA; 10 μM) or lipid peroxidation sensitive probe (C11-BODIPY; 2 μM) for 25 minutes. Finally, the fluorescence signal of each group was detected by flow cytometry (FC500, Beckman). To detect the generated ROS or lipid peroxidation by confocal laser microscopy, cells were stained with DCFH-DA (10 μM) or C11-BODIPY (2 μM) for 25 minutes. The cell nucleus was revealed by staining with Hoechst 33342 for 15 minutes. Fluorescence images of each group were observed by confocal laser scanning microscopy (DFC 7000T, Leica, Germany).

**TEM imaging of arsenene nanosheets treated cells**

U2OS-Her2(+) cells (1×10^6^ cells/dish) were seeded into cell Petri dishes and cultured overnight. The cells were treated with medium containing 10 μg/mL of Her2-ANs and incubated for different times. Subsequently, the cells were washed, fixed, stained, dehydrated, embedded and sliced before being observed by TEM (Talos L120C, Thermo Scientific, USA).

**SEM imaging of arsenene nanosheets treated cells**

U2OS-Her2(+) cells (6×10^4^ cells/well) were seeded into 24-well plates containing round coverslip and cultured overnight. The cells were treated with medium containing 10 μg/mL of Her2-ANs and incubated for different times. Subsequently, the cells were washed, fixed, dehydrated and dried before being observed by SEM (GeminiSEM300, Zeiss, Germany).

**Cell death assay**

U2OS-Her2(+) or U2OS/CDDP cells (1×10^5^ cells/well) were seeded into 12-well plates and cultured overnight. The cells were treated with medium containing nanosheets loaded with cisplatin (PBS, CDDP, Her2-ANs or Her2-ANS@CDDP) and incubated for 20 hours. Cell death was detected with the Annexin V-FITC/PI Double Stain Assay Kit (BB-4101, Bestbio, China) according to the manufacturer's instructions. Finally, the mortality of each group of cells was detected by flow cytometry (BD FACSC anto, BD Biosciences, USA).

**Determination of Pt content within the cell and Pt bound to DNA**

U2OS or U2OS/CDDP cells (3×10^5^ cells/well) were seeded into 6-well plates and cultured overnight. Cells were treated with media containing CDDP or Her2-ANS@CDDP (CDDP: 2 μg/mL) for 8 hours. The cells were then washed three times with PBS and collected into centrifuge tubes. The cells in the centrifuge tubes were frozen overnight at -80°C and then lysed with aqua regia at 95°C for 3 hours. The intracellular Pt content was determined by ICP-MS (NexION 5000, Perkin Elmer, USA). To measure the amount of Pt bound to DNA, cellular DNA was extracted using a DNA extraction kit (DP304, TIANGEN, China) according to the manufacturer's instructions. The amount of Pt bound to DNA was determined by ICP-MS.

**Detection of cellular DNA damage**

U2OS/CDDP cells (6×10^4^ cells/well) were seeded into 24-well plates containing round coverslip and cultured overnight. The cells were treated with medium containing nanosheets loaded with cisplatin (PBS, CDDP, Her2-ANs or Her2-ANS@CDDP) for 20 hours. The cells were incubated overnight with primary antibody of γH2A.X (381558, 1:100, ZenBio, China) after fixation, permeabilization and blocking procedures. The nuclei were stained with DAPI for 5 minutes. Finally, the fluorescence images of each group were observed by confocal laser scanning microscopy (DFC 7000T, Leica, Germany).

**Colony formation assay**

143B-Her2(+) cells (7×10^2^ cells/well) were seeded into 6-well plates and cultured for 24 hours. The cells were treated with medium containing nanosheets loaded with cisplatin (PBS, CDDP, Her2-ANs or Her2-ANS@CDDP) for 20 hours. After treatment, the cells were continued to be cultured with fresh medium for 8 days. After fixation with paraformaldehyde for 15 minutes, the colonies were stained with 1% crystal violet for 30 minutes.

**Animal tumor models**

SPF-grade BALB/c nude mice (male, 3-4 weeks old, 16-18 g) used to construct animal tumor models were purchased from Gempharmatech Co., Ltd. (Nanjing, China). 4×10^6^ 143B-Her2(+) cells were resuspended in 100 μL of PBS and injected subcutaneously into the axilla of the right forelimb of BALB/c nude mice. All animal experiments were performed in accordance with the National Institutes of Health Animal Care Guidelines and were approved by the Animal Experimentation Ethics Committee of Anhui Medical University (Hefei, China) (PZ-2022-035).

**In vivo antitumor study**

To evaluate the *in vivo* antitumor effect of ANs, nude mice with a tumor volume of approximately 250 mm^3^ were randomly divided into five groups (6 mice per group) and injected with PBS, CDDP, ANs, Her2-ANs and Her2-ANs@CDDP (CDDP: 1 mg/kg, As ions: 2 mg/kg) via tail vein, respectively. Mouse body weight and tumor volume were recorded every 2 days from the beginning of treatment. Tumor volume (V) was calculated by the formula V=0.5×L×W^2^, in which L and W refer to the length and width of the tumor, respectively. The relative tumor suppression rate (r_14_) was calculated by dividing the difference between the absolute tumor growth of the control and treated groups by the absolute tumor growth of the control group. Absolute tumor growth for each group was the difference between tumor volume on day 14 and day 0. After two weeks, the mice were euthanized and their blood, organs, and tumor tissue were harvested for subsequent experiments.

**Detection of ROS and lipid peroxide production in vivo**

After two weeks, the harvested tumor tissues from each group were ground to obtain cell suspensions. The cell suspension was filtered with a sieve (200 mesh) to obtain a single-cell suspension. Subsequently, the cells were washed twice with PBS and stained with ROS or lipid peroxide probes as described above.

**Analysis of tumor tissue section**

Apoptosis of tumor cells was assessed using the TUNEL assay kit (B014, ebiogo, China) according to the manufacturer's instructions. To assess the proliferation of tumor cells, tumor sections were immunostained with an antibody against Ki67 (MAB-0672, 1:300, MXB Biotechnology, China). To assess angiogenesis in the tumor, tumor sections were immunofluorescence stained with an antibody against CD31 (77699S, 1:300, Cell Signaling Technology, USA). To assess the GPX4 activity of tumor cells, tumor sections were immunostained with an antibody against GPX4 (R24461, 1:200, ZenBio, China).

**In vivo distribution test**

To investigate the biodistribution of ANs and Her2-ANs, tumor-bearing nude mice were administrated with ANs and Her2-ANs via intravenous injection (n = 3). The heart, liver, spleen, lung, kidney, and tumor were collected and weighed at 24 hours, followed by typical tissue nitrification with aqua regia. Then, an ICP-MS analysis was performed to accurately determine the content of arsenic.

**In vivo biosafety assessment**

Two weeks later, blood from the mice was collected for hematological and blood biochemical tests. Hematological indicators were tested by an automated five-classification blood cell analyzer (BC-5000, Mindray, China). Blood biochemistry was measured by an automated biochemistry analyzer (Catalyst One, IDEXX Laboratories, USA). To evaluate the systemic safety of the nanosheets, the main organs of the mice were analyzed by HE staining.

**Statistical analysis**

Data were expressed as mean ± standard deviation. Differences between the groups were compared using one-way ANOVA. *p<0.05 and **p<0.01 were considered the differences to be statistically significant.

**Additional data**

**Fig. S1.** (A) UMAP visualization of cells analyzed by scRNA-seq and integrated across osteosarcoma derived from a single patient. Clusters were annotated for their cell types as predicted using canonical markers. (B) Log-normalized expression of ERBB2 (Her2).

**Fig. S2.** U2OS (A) and 143B (B) cells were sorted by flow cytometry into Her2(+) and Her2(-) subpopulations.

**Fig. S3.** (A) TEM image of raw arsenic powder. (B) AFM image of raw arsenic powder. (C) Corresponding height profiles along the white lines. (D) UV−Vis spectrum of the raw arsenic powder.

**Fig. S4.** (A) FTIR spectrum of the ANs and Her2-ANs. (B) Raman spectrum of the ANs and Her2-ANs. (C) Raman spectrum of raw arsenic powder. (D, E) The corresponding elemental mapping (D) and EDS (E) of Her2-ANs. (F) The size distributions of the ANs and Her2-ANs. (G) Fluorescence signal of ANs, ANs-FITC and Her2-ANs-FITC.

**Fig. S5.** TEM images of Her2-ANs at different pH values and different GSH concentrations.

**Fig. S6.** GSH-depleting effects of Her2-ANs *in vitro*.

**Fig. S7.** (A, B) Flow cytometric analysis of cell death in U2OS-Her2(+) cells after various treatments (CDDP: 2 μg/mL; As ions: 10 μg/mL). (Values are presented as means ± sd, n = 3, *P < 0.05, **P < 0.01.) (C) Relative viabilities of U2OS-Her2(+), U2OS-Her2(-) and AML-12 cells after incubation with Her2-ANs at different concentrations. (Values are presented as means ± sd, n = 3, *P < 0.05, **P < 0.01.)

**Fig. S8.** (A) Intracellular uptake of ANs, Her2-ANs and raw arsenic powder in the U2OS-Her2(+) cells was measured by ICP-MS. (B) The viability of U2OS-Her2(+) cells treated with Her2-ANs after rescue with various inhibitors, including GSH, ferrostatin-1, NAC, DFOM, and Z-VAD-FMK. (Values are presented as means ± sd, n = 3, *P < 0.05, **P < 0.01.)

**Fig. S9.** (A, B) Flow cytometric analysis of the production of ROS (A) and lipid peroxide (B) in U2OS-Her2(+) cells after treatment with Her2-ANs. (C, D) Confocal laser scanning microscopy imaging of ROS (C) and lipid peroxide (D) in U2OS-Her2(+) cells after treatment with Her2-ANs. Scale bar: 100 μm.

**Fig. S10.** (A, B) Statistical analysis of Western blot results for GPX4 expression in U2OS-Her2(+) cells after treatment with Her2-ANs at different concentrations (A) and times (B). (Values are presented as means ± sd, n = 3, *P < 0.05, **P < 0.01.)

**Fig. S11.** Cytotoxicity of CDDP against the U2OS and U2OS/CDDP cells was determined by CCK-8 assay.

**Fig. S12.** Proliferation ability of the 143B-Her2(+) cells with different treatments was detected by a colony formation assay.

**Fig. S13.** Accumulation of As ions in the major organs or tumors of tumor-bearing nude mice evaluated by ICP-MS at 24 hours. (Values are presented as means ± sd, n = 3, *P < 0.05, **P < 0.01.)

**Fig. S14.** Hematological parameters of tumor-bearing mice after intravenous injection of PBS, CDDP, ANs, Her2-ANs and Her2-ANs@CDDP for 2 weeks.

**Fig. S15.** Total protein (TP), albumin (ALB), globulin (GLB), total bilirubin (TBIL), alanine aminotransferase (ALT), aspartate aminotransferase (AST), lactate dehydrogenase (LDH), and urea values of tumor-bearing mice after intravenous injection of PBS, CDDP, ANs, Her2-ANs and Her2-ANs@CDDP for 2 weeks.

**Fig. S16.** Histopathological examination of the major organs of tumor-bearing mice after intravenous injection of PBS, CDDP, ANs, Her2-ANs and Her2-ANs@CDDP for 2 weeks. Scale bar: 100 μm.

**Fig. S17.** (A) The analysis of the hemolysis rate. (B) Pictures of Her2-ANs incubated with blood for 8 hours.

**Table S1.** Details of the osteosarcoma samples used for single-cell sequencing.

| Patient number | Gender | Age (years) | Site of tumor occurrence | Pathological diagnoses |
| --- | --- | --- | --- | --- |
| 1 | Female | 12 | Humerus | Osteosarcoma |
| 2 | Female | 14 | Humerus | Osteosarcoma |
| 3 | Male | 7 | Femur | Osteosarcoma |
| 4 | Male | 19 | Femur | Osteosarcoma |
| 5 | Female | 55 | Femur | Osteosarcoma |

**Table S2.** Details of the osteosarcoma samples used for immunohistochemistry.

| Patient number | Gender | Age (years) | Site of tumor occurrence | Pathological diagnoses |
| --- | --- | --- | --- | --- |
| 1 | Female | 12 | Humerus | Osteosarcoma |
| 2 | Female | 14 | Humerus | Osteosarcoma |
| 3 | Male | 7 | Femur | Osteosarcoma |
| 4 | Male | 19 | Femur | Osteosarcoma |
| 5 | Female | 55 | Femur | Osteosarcoma |
| 6 | Male | 13 | Tibia | Osteosarcoma |
| 7 | Male | 14 | Femur | Osteosarcoma |
| 8 | Female | 14 | Femur | Osteosarcoma |
